# Deletion of homologs of the SREPB pathway results in hyper-production of cellulases in *Neurospora crassa* and *Trichoderma reesei*

**DOI:** 10.1186/s13068-015-0297-9

**Published:** 2015-08-19

**Authors:** Morgann C Reilly, Lina Qin, James P Craig, Trevor L Starr, N Louise Glass

**Affiliations:** Department of Plant and Microbial Biology, University of California, Berkeley, CA 94720 USA; The Energy Biosciences Institute, University of California, Berkeley, CA 94720 USA

**Keywords:** *Neurospora crassa*, Secretion, Cellulase, Cellulose, *Trichoderma reesei*, Lignocellulose, SREBP, Dsc E3 ligase

## Abstract

**Background:**

The filamentous fungus *Neurospora crassa* efficiently utilizes plant biomass and is a model organism for genetic, molecular and cellular biology studies. Here, a set of 567 single-gene deletion strains was assessed for cellulolytic activity as compared to the wild-type parental strain. Mutant strains included were those carrying a deletion in: (1) genes encoding proteins homologous to those implicated in the *Saccharomyces cerevisiae* secretion apparatus; (2) genes that are homologous to those known to differ between the *Trichoderma reesei* hyper-secreting strain RUT-C30 and its ancestral wild-type strain; (3) genes encoding proteins identified in the secretome of *N. crassa* when cultured on plant biomass and (4) genes encoding proteins predicted to traverse the secretory pathway.

**Results:**

The 567 single-gene deletion collection was cultured on crystalline cellulose and a comparison of levels of secreted protein and cellulase activity relative to the wild-type strain resulted in the identification of seven hyper-production and 18 hypo-production strains. Some of these deleted genes encoded proteins that are likely to act in transcription, protein synthesis and intracellular trafficking, but many encoded fungal-specific proteins of undetermined function. Characterization of several mutants peripherally linked to protein processing or secretion showed that the hyper- or hypo-production phenotypes were primarily a response to cellulose. The altered secretome of these strains was not limited to the production of cellulolytic enzymes, yet was part of the cellulosic response driven by the cellulase transcription factor CLR-2. Mutants implicated the loss of the SREBP pathway, which has been found to regulate ergosterol biosynthesis genes in response to hypoxic conditions, resulted in a hyper-production phenotype. Deletion of two SREBP pathway components in *T. reesei* also conferred a hyper-production phenotype under cellulolytic conditions.

**Conclusions:**

These studies demonstrate the utility of screening the publicly available *N. crassa* single-gene deletion strain collection for a particular phenotype. Mutants in a predicted E3 ligase and its target SREBP transcription factor played an unanticipated role in protein production under cellulolytic conditions. Furthermore, phenotypes similar to those observed in *N. crassa* were seen following the targeted deletion of orthologous SREBP pathway loci in *T. reesei*, a fungal species commonly used in industrial enzyme production.

**Electronic supplementary material:**

The online version of this article (doi:10.1186/s13068-015-0297-9) contains supplementary material, which is available to authorized users.

## Background

*Neurospora crassa* was first described following the investigation of a mold infestation in French bakeries during the 1840s [1]. The organism can be readily isolated from the environment, often in association with burned grasses, sugar cane or trees [2, 3]. *N. crassa* has been a well-established model system in the laboratory for over a century [4] and as a result there are numerous tools available for its study, including a nearly complete single-gene deletion strain collection [5, 6].

Observations of enzyme activities related to the ability of *Neurospora* to utilize plant biomass were first reported in the 1950s [7]. A systems biology analysis of the *N. crassa* transcriptome and secretome associated with utilization of plant biomass and cellulose revealed a robust enzymatic response [8]. A regulon of 212 genes that are transcriptionally induced in *N. crassa* in the presence of a high-purity crystalline cellulose (Avicel) as compared to starvation or rich media culture conditions has been defined [9]. The secretion of 38 proteins, as identified by mass spectrometry, was also associated with these cellulosic culture conditions [8]. Almost 40% of the total secretome by weight is attributable to a single cellulolytic enzyme, cellobiohydrolase-1 (CBH-1 or NCU07340) [10]. Additional studies exploring *Neurospora’s* response to xylan [11] and pectin [12] have furthered our understanding of a filamentous fungus’ systematic response to plant biomass.

In the current model, under conditions of starvation, *N. crassa* produces low levels of hydrolytic enzymes capable of digesting a broad range of nutrient sources. When these ‘scout’ enzymes encounter plant cell wall material, specific breakdown products are generated that signal to *N. crassa* the presence of an utilizable carbon source [13, 14]. In the case of cellulose, the main byproducts are cellodextrins, which can induce the production of lignocellulose-degrading enzymes [15]. The *N. crassa* transcriptional response to cellulose is primarily controlled by the transcription factors CLR-1 (NCU07705) and CLR-2 (NCU08042) [9], which regulate the production of cellulases and accessory proteins. Studies have shown that the mis-expression of CLR-2 is sufficient, even under non-inducing conditions, for the production of cellulases and some hemicellulases [16].

The *N. crassa* enzymes involved in extracellular plant cell wall deconstruction must traverse the secretory pathway in order to be transported across the fungal plasma membrane. Transcription of genes encoding translocation machinery that traffic mRNA/proteins destined for the endoplasmic reticulum is significantly increased during growth of *N. crassa* on Avicel [9, 12], suggesting a specific role for components of the secretory pathway in the trafficking of proteins needed for plant cell wall deconstruction and utilization. In the yeast *Saccharomyces cerevisiae*, the secretory apparatus has been extensively characterized [17] and is considered a model for eukaryotic protein secretion. However, recent studies in the filamentous fungal species *Trichoderma reesei*, *Aspergillus* sp. and *Neurospora crassa* have begun to highlight not only the similarities, but also the differences in function of the secretory pathway in filamentous fungi as compared to *S. cerevisiae* [18–21]. As a result, investigations into protein trafficking in filamentous fungal species are needed to understand the secretion of plant biomass degrading enzymes by these multicellular organisms.

In this study, strains of *N. crassa* with single-gene deletions in loci thought likely to play a role in cellulase activity or secretion were assayed for protein production and enzyme activity when grown on a cellulosic substrate (Avicel). Initial work with several hyper-production deletion strains has implicated genes encoding components of the Sterol Regulatory Element Binding Protein (SREBP) pathway, which has not been previously linked to protein secretion. Deeper characterization of these mutants will enhance our knowledge of the underpinnings and mechanisms associated with the secretion of proteins involved in plant biomass deconstruction and utilization of complex carbon sources by filamentous fungi, which can be further manipulated in industrial settings to enable increased protein production.

## Results

### Screening of deletion strains for protein secretion and cellulase activity

Single-gene deletion strains were selected from the *N. crassa* full genome deletion collection available through the Fungal Genetics Stock Center [5, 6] based on four criteria. First, genes encoding proteins that potentially function in the secretory apparatus (such as SNARE proteins involved in intracellular vesicle docking and fusion, GTPases that play a role in vesicle formation, and GPI-anchored or myristoylated proteins that link to membranes) were selected based on homology to known proteins in *S.cerevisiae* (http://www.yeastgenome.org). This process led to the inclusion of 103 deletion strains (Additional file 1: Table S1; Category A). Second, *N. crassa* homologs to genes in *T. reesei* that varied in DNA sequence between the hyper-cellulolytic RUT-C30 strain and its parental wild isolate QM6a [22, 23] were identified. From this analysis, another 47 deletion strains were included (Additional file 1: Table S1; Category B). Third, deletion strains for known and predicted cellulases and hemicellulases of *N. crassa* as well as those proteins identified in the *Neurospora* secretome when grown on either crystalline cellulose (Avicel) or ground *Miscanthus* x *gigantaeus* [8] were included (34 deletion strains; Category C; Additional file 1: Table S1). Fourth, additional genes encoding secreted proteins were identified by evaluating the predicted *Neurospora* proteome via SignalP (http://www.cbs.dtu.dk/services/SignalP) and TargetP (http://www.cbs.dtu.dk/services/TargetP) algorithms. The 596 loci with high scores from both programs (SignalP score >0.5 and TargetP score = 1) were considered likely to encode proteins that traversed the secretory pathway. Deletion strains were available for 384 of these identified genes (Additional file 1: Table S1; Category D); the remaining 212 loci either had no deletions or were deposited as a heterokaryon. The combination of all four categories led to a total of 567 strains (Additional file 1: Table S1), with almost half carrying deletions in genes that encode ‘hypothetical proteins’ (http://www.broadinstitute.org/annotation/genome/neurospora/MultiHome.html).

Conidial suspensions from each of the 567 deletion strains listed in Additional file 1: Table S1 were randomly arrayed in deep-well, 96-well plates. For each plate, duplicate wells of the parental strain for the deletion collection (FGSC 2489 [5]), a positive control strain (ΔNCU06650—a strain previously identified as a hyper-producer of cellulases [8, 24]) and a negative control strain (ΔNCU07340 or Δ*cbh*-*1*—a strain with limited cellulolytic activity due to the loss of cellbiohydrolase-1 [8]) were included. Strains were propagated on plugs of sucrose-based Vogel’s minimal medium (VMM [25]) agar in deep-well, 96-well plates and conidia harvested from these plates were used to inoculate cellulosic medium (Avicel in combination with Vogel’s salts) in deep-well, 24-well plates. After 4 days of growth, the supernatant from each culture was harvested and assayed for total protein level and enzyme activity (endoglucanase and Avicelase assays [8]).

Each 96-well plate of deletion and control strains was processed independently and in triplicate and the results of the protein and enzyme activity assays were evaluated relative to the internal control strains on each plate (FGSC 2489, ΔNCU06650, Δ*cbh*-*1*). Strains that consistently ranked in the top or bottom 20% of each plate for secreted protein and cellulolytic activity were subsequently arrayed in glycerol stocks in new deep-well, 96-well plates along with the same three control strains. If available, genetically identical deletion strains in both the *mat A* and *mat a* mating type background were included. Strains were re-evaluated for secreted protein and enzyme activity levels as compared to the included control strains, again in triplicate.

In addition to culturing on Avicel, each strain that came through the primary screen was also inoculated into sucrose-based VMM to assess overall growth phenotypes. Strains that conidiated poorly, showed limited growth in VMM, did not have matching phenotypes between *matA* and *mata* strains or did not demonstrate consistent results between experimental replicates were discarded. From these secondary screens, 25 strains of interest were identified (Table 1): seven deletion strains that showed approximately double the wild-type level of secreted protein and associated cellulolytic activity (hyper-production strains) and 18 deletion strains that showed roughly less than half the amount of protein secretion and cellulolytic activity of wild type (hypo-production strains).

**Table 1:** Single-gene deletion strains of *N. crassa* with altered cellulase production compared to the parental wild-type strain

Phenotype	Deleted locus	Category^a^	Fold change^b^	Gene annotation	Conservation	*S. cerevisiae* homolog^c^
Increased secreted cellulases	NCU00541	B	1.9	Hypothetical protein	Asco	None
	NCU01291	D	2.5	Hypothetical protein	Asco	None
	NCU02336	D	1.9	Capsule associated protein	Asco	None
	*NCU03459*	*D*	*2.9*	*UBA domain*-*containing protein Ucp14*	*Asco, Basidio*	*DSC2* (4.7e−17)^d^
	*NCU03740*	*D*	*4.2*	*Hypothetical protein*	*Asco*	*TUL1* (1.6e−28)
	NCU06650	D	2.3	Secretory phospholipase A2	Asco, Gram^+^	None
	NCU07788	B	1.9	Colonial-26	Asco	YFL052W (9.8e−16)
Decreased secreted cellulases	NCU00566	A	0.4	Synaptobrevin	Asco, Basidio	*SNC2* (1.2e−20)
	*NCU00600*	*A*	*0.2*	*Rho*-*type GTPase*-*3*	*Asco*	*RHO3* (6.7e−66)
	NCU00761	D	0.6	Triacylglycerol lipase	Asco, Basidio	None
	NCU01739	D	0.3	Hypothetical protein	Asco, Basidio	None
	NCU02017	B	0.0	All development altered-2	Asco, Basidio	*NCB2* (3.7e−26)
	NCU02152	B	0.4	RRM domain-containing protein	Asco, Basidio	None
	NCU02182	B	0.7	Hypothetical protein	Asco, Basidio	None
	NCU04593	D	0.6	Lcc9	Asco	*FET5* (2.9e−100)
	NCU04952	C	0.2	Glycosylhydrolase family 3–4	Asco, Basidio	None
	*NCU05213*	*A*	*0.2*	*Hypothetical protein*	*Asco*	*TRS85* (4.8e−18)
	NCU05547	D	0.5	Hypothetical protein	Asco	None
	NCU05720	A	0.6	Chitin synthase export chaperone	Asco, Basidio	*CHS7* (1.6e−62)
	NCU05890	D	0.3	Hypothetical protein	Euk	None
	NCU06086	D	0.1	Regulatory protein suaprga1	Asco, Basidio	*MAM33* (2.3e−31)
	NCU06703	D	0.4	Mating pheromone-induced death-1	Asco	*MID1* (3.4e−28)
	NCU07062	A	0.3	Serine/threonine protein kinase-49	Asco, Basidio	*SCH9* (4.9e−48)
	NCU08339	D	0.4	Endosomal P24B protein	Asco	*EMP24* (9.8e−49)
	NCU08909	C	0.6	Beta-1,3-glucanosyltransferase	Asco	*GAS1* (3e−111)

*Neuro* *Neurospora* genus, *Asco* ascomycete fungi, *Basidio* basidiomycete fungi, *Gram* ^*+*^ Gram-positive bacteria, *Euk* higher eukaryotes.

^a^Category A: Proteins potentially involved in cellular secretion apparatus (e.g., tether and SNARE proteins, GTPases, GPI-anchored and myristoylated proteins). Category B: Homologs of *T. reesei* genes that vary between wild type and the hyper-producing RUT-C30 strain as identified in [22, 23]. Category C: Known and predicted cellulases, hemicellulases and other proteins secreted in response to cellulose as identified in [8]. Category D: Proteins that traverse the secretory pathway as predicted using the SignalP (score >0.5) and TargetP (score = 1) algorithms.

^b^Fold-change relative to wild type for secreted protein as measured by Bradford assay (*n* = 6). When deletions were available in both the *mat A* and *mat* *a* mating type backgrounds, the two values were averaged.

^c^Defined as nearest homolog with an *E* value <1e−10 using BlastP.

^d^Only protein for which reciprocal BlastP using *S. cerevisiae* sequence against *N. crassa* genome does not return original locus; homology with Ucp14p of *Schizosaccharomyces pombe.*

### Identification of hyper- and hypo-production strains

The 25 hyper- and hypo-production strains were distributed across the four categories (A–D) originally used to identify the strains included in the screen (Table 1). The genes deleted in each of these strains were conserved across the Ascomycota and many were also maintained throughout the Basidiomycota (Table 1). However, 11 of the deleted loci did not have identifiable homologs (<1e−10) in the *S. cerevisiae* genome, suggesting that their function may be specific to the biology of filamentous fungi.

The Category A deletion strains in Table 1, which are predicted to encode elements of the secretory apparatus, showed reduced levels of secreted protein and cellulase activity as compared to the wild-type strain. These encoded proteins were homologous to known secretion components in *S. cerevisiae*, such as NCU00566 (*SNC2* homolog), NCU00600 (*RHO3* homolog), NCU05213 (*TRS85* homolog), NCU05720 (*CHS7* homolog) and NCU08339 (*EMP24* homolog).

The genes identified in Category B—*N. crassa* homologs of genes carrying mutations in the *T. reesei* RUT-C30 hyper-production strain [22, 23]—were from a variety of functional categories. Mutations in two genes, one encoding a hypothetical protein (NCU00541) and one encoding the transcription factor COL-26 (NCU07788) [5], showed increased secretion and cellulase activity. *col*-*26* is a homolog to *bglR* in *T. reesei* [26], which has been implicated in the regulation of β-glucosidase genes. In *N. crassa*, *col*-*26* is important for nutrient sensing and genetically interacts with *vib*-*1* and *cre*-*1*, which are important for utilization of cellulose and carbon catabolite repression, respectively [27]. The remaining three Category B mutants showed decreased cellulase activity and secreted protein levels, including the deletion of the transcription factor ADA-2 (NCU02017) [5], a locus predicted to encode an RNA-binding protein (NCU02152) and one gene encoding a hypothetical protein (NCU02182).

Two of the identified hypo-production strains carried deletions in genes encoding proteins present in the *N. crassa* cellulolytic secretome (Table 1; Category C). One, NCU08909 (*gel*-*3*), encodes a GPI-anchored β-1,3-glucanosyltransferase implicated in cell wall biosynthesis [28]. The other, NCU04952 (*gh3*-*4*), encodes a glycosylhydrolase family 3 member that functions as an extracellular β-glucosidase [15].

Thirteen strains that displayed a secretion phenotype contained a deletion of a gene encoding a protein predicted to traverse the secretory pathway based on its scores using the SignalP and TargetP algorithms (Table 1; Category D). Five of the Category D mutants encode hypothetical proteins, all of which lack significant homology to genes in *S. cerevisiae*. The remaining eight genes showed sequence homology to proteins with identified function in *S. cerevisiae* or other fungal species (Table 1). Of the 13 Category D mutants in Table 1, only three have been previously characterized in *N. crassa*: NCU06650, which encodes a phospholipase A [8, 24], NCU06703 (*mid*-*1*), which encodes a mechanosensitive calcium channel [29] and NCU06086, which is predicted to be part of the *N. crassa* mitochondrial proteome [30].

### Phenotypic characterization of select hyper- and hypo-production strains

For proof of principle, we focused our studies on four mutants: two showing consistently increased protein secretion and cellulase activity from the hyper-production mutants (ΔNCU03459 and ΔNCU3740) and two that showed consistently decreased protein secretion and cellulase activity relative to the other hypo-production mutants (ΔNCU00600 and ΔNCU05213) (Table 1; italics). The targeted proteins in each of these four deletion mutants are predicted to play various roles in protein processing and secretion. NCU03459 is annotated as encoding an ubiquitin binding associated (UBA) protein and has sequence similarity to Dsc2p (formerly YOL073C) in *S. cerevisiae* and Dsc2 (formerly Ubc14) in *S. pombe*, both of which function in association with a Golgi E3 ligase complex [31, 32]. The second mutant associated with hyper-production, ΔNCU03740, has significant similarity to ubiquitin ligases, most closely related to that of Tul1p in *S. cerevisiae* and Dsc1 in *S. pombe*. One of the mutants displaying a hypo-production phenotype, ΔNCU00600, encodes a protein that is similar to a GTPase, Rho3p, which is involved in polarized secretion in *S. cerevisiae* [33, 34]. The remaining mutant with a hypo-production phenotype, ΔNCU05213, shows similarity to Trs85p, which is part of the TRAPP-III complex involved in ER-Golgi trafficking and autophagy in *S. cerevisiae* [35].

On sucrose-based VMM agar, the ΔNCU00600 (Δ*rho*-*3*) and ΔNCU05213 (Δ*trs85*) hypo-production mutants grossly resembled FGSC 2489 (wild type) in morphology (Additional file 2: Figure S1A), although under higher magnification these two mutants showed a hyper-branching hyphal structure (Additional file 2: Figure S1B). For the hyper-production strains, both ΔNCU03740 (Δ*tul*-*1*) and ΔNCU03459 (Δ*dsc*-*2*) showed reduced aerial hyphae compared to wild type (Additional file 2: Figure S1A), but when examined at a higher magnification, these two hyper-production strains displayed growth and hyphal branching patterns that were similar to the wild-type parent (Additional file 2: Figure S1B). On Avicel agar, all strains, including the wild-type strain, showed a reduced hyphal diameter as compared to their growth on VMM agar (Additional file 2: Figure S1C). However, obvious differences in hyphal tip morphology or internal organelle structure in the hyphae of the four mutant strains were not observed using the plasma membrane endocytic dye FM4-64 or an ER dye (ER-Tracker Red) under either VMM or Avicel conditions (Additional file 2: Figure S1C and D). All four strains appeared to grow equally well when cultured in VMM, xylan and Avicel media, with the possible exception of Δ*rho-3* mutant on Avicel (Additional file 2: Figure S1E).

### Protein secretion and enzyme activity in the hyper- and hypo-production strains

**Fig. 1:**
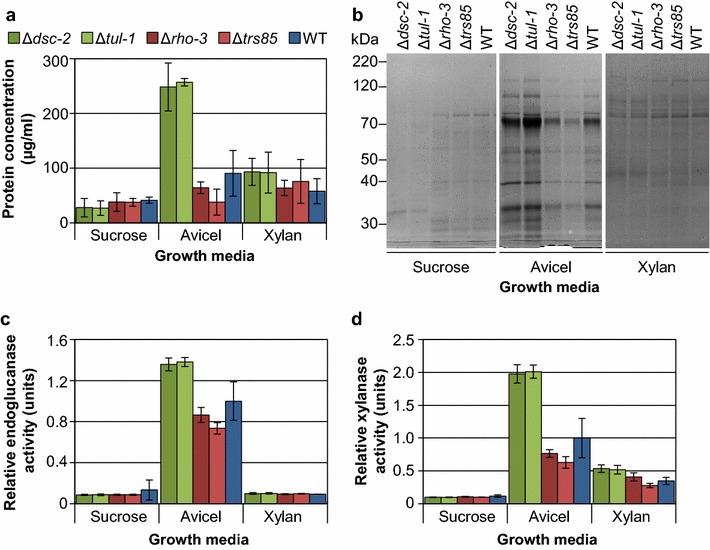
Secretion phenotypes are cellulose-specific. **a** Total secreted protein as measured by Bradford assay. **b** SDS-PAGE analysis of total secreted protein, equal volumes loaded. **c**, **d** Endoglucanase and xylanase activities, respectively, as measured using dye-release assays. Conidia from wild type and the deletions strains were inoculated directly into growth media containing sucrose (24 h), Avicel (96 h) or xylan (48 h). Protein and enzyme activity levels in the culture supernatants relative to the parental wild-type strain under each condition were analyzed by one-way ANOVA using the Dunnett’s multiple comparisons test. Data drawn from biological replicates (*n* = 4); *error bars* indicate standard deviation.

None of the four selected deletion strains demonstrated a statistically significant difference relative to the wild-type strain in the amount of protein present in culture supernatants when grown in either sucrose- or xylan-based media (Fig. 1a). However, the Δ*dsc*-*2* and Δ*tul*-*1* strains showed significant increases (*p* ≤ 0.0001) in secreted protein levels as compared to wild type when cultured in Avicel, while both the Δ*rho*-*3* and Δ*trs85* strains showed consistently lower secreted protein levels than the parental wild-type strain. When equal volumes of culture supernatants were analyzed by SDS-PAGE, an increased level of all secreted proteins was observed for the Δ*dsc*-*2* and Δ*tul*-*1* mutants compared to wild type under only Avicel conditions (Fig. 1b). In addition, the Δ*rho*-*3* and Δ*trs85* mutants showed reduced levels of secreted proteins under Avicel conditions, though the protein banding pattern remained similar to the wild-type strain. Under xylan conditions, the pattern and levels of proteins observed were similar for all strains (Fig. 1b). When cultured in the presence of sucrose, the composition of the secretome from the Δ*dsc*-*2* and Δ*tul*-*1* mutants differed from that of the wild-type strain, while the secretome of the Δ*rho*-*3* and Δ*trs85* mutants were similar to each other and to the wild-type strain.

Supernatants from the Avicel-grown Δ*dsc*-*2* and Δ*tul*-*1* cultures showed significantly elevated endoglucanase activity levels relative to the wild-type strain (*p* ≤ 0.0001), while the Δ*rho*-*3* and Δ*trs85* mutants were lower (Fig. 1c). Endoglucanase activity was virtually undetectable for all strains cultured in either VMM or xylan medium, indicating that a cellulosic inducer was still required by each of these strains to produce cellulases. In wild-type supernatants, hemicellulolytic (xylanase) activity is detectable from cultures grown on xylan or Avicel. The latter is primarily due to the induction of several xylanases by the cellulose-specific transcription factor CLR-2 [9, 16], but also the minor contamination of commercially available Avicel with hemicellulose [27]. When cultured on Avicel, xylanase activity in the Δ*dsc*-*2* and Δ*tul*-*1* strains was also significantly higher (*p* ≤ 0.0001) than that for the wild-type strain, whereas the Δ*rho*-*3* and Δ*trs85* strains showed reduced xylanase activity (Fig. 1d). Although no major difference in protein secretion levels was apparent when the mutant strains were cultured on xylan (Fig. 1a), the level of xylanase activity in the hyper-production strains Δ*dsc*-*2* and Δ*tul*-*1* was slightly higher relative to the wild-type strain (*p* ≤ 0.01).

**Fig. 2:**
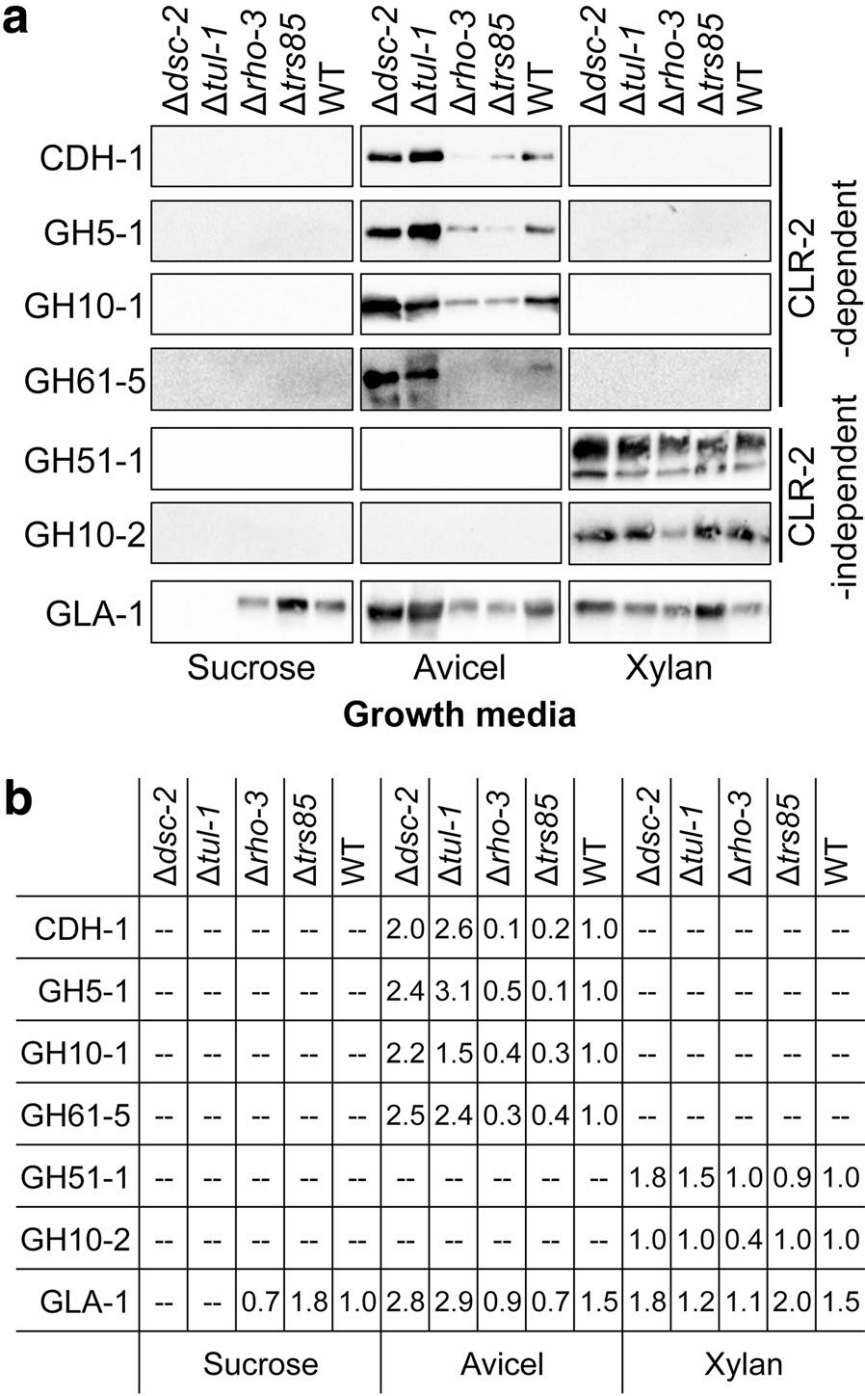
Secretion phenotypes are not limited to cellulases. **a** Immunoblot analysis of total secreted protein from experiment outlined in Fig. 1. Equal volumes of culture supernatant were loaded and separated by electrophoresis on 10% polyacrylamide gels, then transferred to nitrocellulose membranes for antibody detection. Primary antibodies were directed against the following: CDH-1 (cellobiose dehydrogenase), GH5-1 (an endoglucanase), GH10-1 (an endoxylanase), GH61-5 (a polysaccharide monooxygenase), GH51-1 (an arabinofuranosidase), GH10-2 (an endoxylanase) and GLA-1 (glucoamylase). **b** Quantitation of detected proteins. For CDH-1, GH5-1, GH10-1 and GH61-5 values are given relative to WT in Avicel; for GH51-1 and GH10-2 values are given relative to WT in xylan; for GLA-1 values are given as relative to WT in sucrose.

The enzyme activity and protein secretion data (Fig. 1) found that the effects associated with the hyper- and hypo-production phenotype of the deletion mutants were more dramatic when grown on a cellulosic substrate. The production of cellulases in *N. crassa* is directly regulated by the cellulose-responsive transcription factor, CLR-2 [9, 16]. We therefore evaluated protein levels in the mutants using antibodies targeted to enzymes whose production is regulated by CLR-2 under cellulosic conditions, including: cellobiose dehydrogenase (CDH-1; NCU00206), an endoglucanase (GH5-1; NCU00762), an endoxylanase (GH10-1; NCU05924) and a polysaccharide monooxygenase (GH61-5; NCU08760) (Fig. 2). In addition, we used antibodies to enzymes known to be independent of CLR-2 regulation, such as an alpha-l-arabinofuranosidase (GH51-1; NCU02343) and an endoxylanase (GH10-2; NCU08189). We also included an antibody to glucoamylase (GLA-1; NCU01517), an enzyme that is constitutively produced by wild-type *N. crassa* under sucrose, Avicel and xylan growth conditions [36].

Glucoamylase-1 was detected in the wild type, Δ*rho*-*3* and Δ*trs85* supernatants for all of the growth media used (Fig. 2). However, in the hyper-production strains Δ*dsc*-*2* and Δ*tul*-*1*, GLA-1 was undetectable when the mutants were grown under sucrose conditions despite visibly apparent fungal cell biomass (Additional file 2: Figure S1E). When grown on Avicel, the protein levels of the CLR-2-dependent proteins CDH-1, GH5-1, GH10-1 and GH61-5 recapitulated the hyper-production phenotype of the Δ*dsc*-*2* and Δ*tul*-*1* mutants, as well as the hypo-production phenotype of the Δ*rho*-*3* and Δ*trs85* mutants (Fig. 2). This finding was also true for the GLA-1 enzyme under cellulolytic growth conditions. The two CLR-2-independent proteins, GH51-1 and GH10-2, were detected only under xylan conditions. Importantly, neither the hyper-production phenotype of the Δ*dsc*-*2* and Δ*tul*-*1* mutants, nor the hypo-production phenotype of the Δ*rho*-*3* or Δ*trs85* mutants, was recapitulated under xylan growth conditions: GH51-1 was present at roughly equivalent levels for all of the strains tested, while GH10-2 levels were only decreased in the Δ*rho*-*3* mutant. Collectively, these data indicate that the hyper-production phenotype of Δ*dsc*-*2* and Δ*tul*-*1* mutants was only apparent under cellulolytic conditions, but that this phenomenon was not limited to cellulosic enzymes.

### The hyper-production phenotype of the Δ*dsc-2* and Δ*tul-1* mutants is retained on plant biomass

Our data showed that the hyper-production phenotypes of the Δ*dsc*-*2* and Δ*tul*-*1* mutants and the hypo-production phenotype of the Δ*rho*-*3* and Δ*trs85* strains were strongest on crystalline cellulose (Figs. 1a–c, 2). We next evaluated whether these phenotypes were restricted to Avicel or could be observed when *N. crassa* was exposed to other cellulosic substrates, including the soluble cellulose substrate carboxymethyl cellulose (CMC) and plant biomass (*Miscanthus* x *giganteus*). For these studies, conidia from the four mutant strains were first inoculated into VMM for 48 h to accumulate biomass before being transferred to either Avicel, CMC or ground *Miscanthus* for 48 h. This approach was used to eliminate growth rate differences between the Avicel, CMC and *Miscanthus*-grown cultures, as well as any conidial germination differences, such as those previously reported for *N. crassa* grown in Avicel versus *Miscanthus* media [8].

**Fig. 3:**
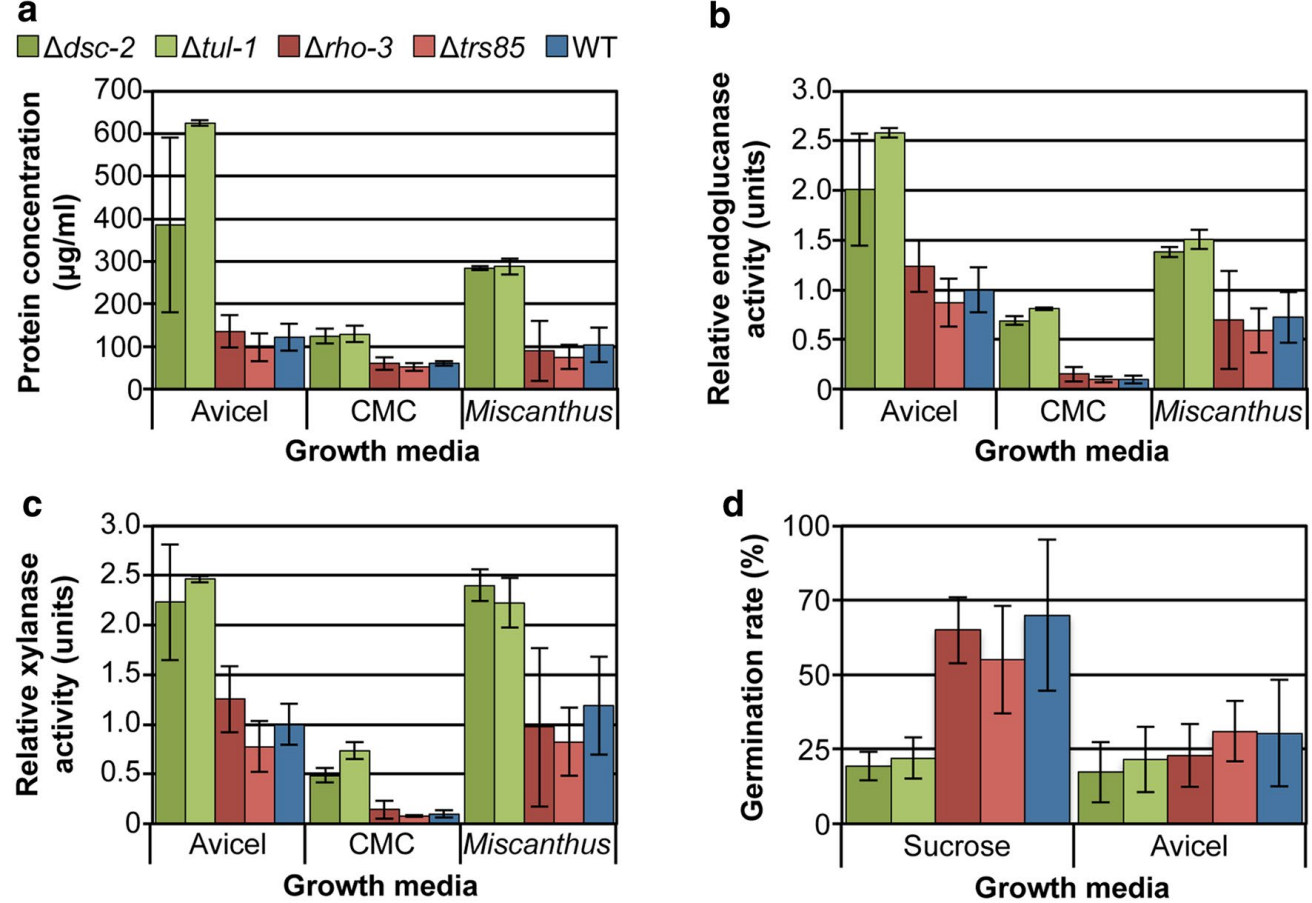
Secretion phenotypes are maintained on cellulosic substrates. **a** Total secreted protein. **b**, **c** Endoglucanase and xylanase activities, respectively. Conidia from wild type and the mutant strains were first inoculated into VMM (48 h) and the resulting biomass subsequently switched to Avicel, CMC, or *Miscanthus* (48 h). **d** Germination frequency. Conidia were cultured in sucrose (4 h) or Avicel broth (8 h) and the numbers of germinated conidia were evaluated by light microscopy. Statistical analyses for protein and enzyme activity levels were relative to WT within each condition (*n* = 3). *Error bars* indicate standard deviation.

The cellulase and protein hyper-production phenotype of the Δ*dsc*-*2* and Δ*tul*-*1* mutants was recapitulated when these mutants were switched into Avicel, CMC or *Miscanthus* media (*p* ≤ 0.01; Fig. 3). However, the reduced protein secretion observed in the Δ*trs85* and Δ*rho*-*3* mutants when conidia were directly inoculated into Avicel (Fig. 1) was not recapitulated when mycelia of these mutants grown on VMM was switched to Avicel, CMC or *Miscanthus*, although the Δ*trs85* mutant consistently trended below that of wild type with regard to protein secretion and enzyme activity levels. These observations suggested that the Δ*rho*-*3* and Δ*trs85* mutants might be defective for germination and/or colony establishment when conidia were directly inoculated into Avicel, but that this phenotype disappeared if fungal biomass was first established in sucrose-based VMM. We therefore evaluated germination frequency of conidia from the Δ*dsc*-*2*, Δ*tul*-*1*, Δ*rho*-*3* and Δ*trs85* mutants as compared to the wild-type parental strain in both VMM and Avicel broth. No correlation was found between the hyper- or hypo-production phenotypes and the conidial germination rates of the mutants versus the parental wild-type strain (Fig. 3d). In fact, while the Δ*rho*-*3* and Δ*trs85* hypo-production mutants showed a similar germination rate to the wild-type strain under all conditions, the Δ*dsc*-*2* and Δ*tul*-*1* hyper-production strains germinated much more slowly than the wild-type strain in VMM. From these data, we hypothesize that the hypo-production phenotype identified in the Δ*trs85* and Δ*rho*-*3* mutants is attributable to a reduced establishment of hyphal growth after conidial germination of these mutants under cellulolytic conditions.

In our screen we identified 18 mutants that showed a cellulolytic hypo-production phenotype when conidia from these strains were directly inoculated into Avicel medium (Table 1). We hypothesized that some of these mutants, like Δ*rho*-*3* and Δ*trs85,* might also lose their hypo-production phenotype when the mutants were first allowed to establish fungal biomass under sucrose-based VMM conditions. To test this hypothesis, the hypo-production strains were allowed to generate biomass in VMM and subsequently switched to Avicel medium. The culture supernatants were then evaluated for secreted protein and enzyme activity levels (data not shown). All but a few of the hypo-production mutants (ΔNCU02017, ΔNCU04952 and ΔNCU07062) lost their hypo-production phenotypes when pre-incubated in VMM prior to exposure to Avicel (Table 1). These data suggest that biomass establishment under recalcitrant carbon conditions is a major factor in the reduced secretion phenotype observed in many of the *N. crassa* hypo-production mutants.

### The Δ*dsc-2* and Δ*tul-1* strains maintain their hyper-production phenotype under constitutive CLR-2-driven cellulase production

The hyper-production phenotype of the Δ*dsc*-*2* and Δ*tul*-*1* strains was most prominent when these strains were grown in Avicel medium, but was also maintained in the presence of the cellulosic substrates CMC and *Miscanthus* (Figs. 1, 3). These data suggested that either the phenotype of these mutants was the result of conditions generated by growth on cellulose or was specifically related to the induction and secretion of cellulases that occurs in response to cellulose exposure. To differentiate between these two possibilities, we utilized a *N. crassa* strain in which the transcriptional activator, *clr*-*2*, is expressed in the absence of a cellulose inducer. Mis-expression of *clr*-*2* under non-cellulolytic conditions recapitulates the wild-type *Neurospora* response to cellulose, resulting in the production and secretion of cellulases when the strain is grown in VMM [16]. We therefore crossed the Δ*dsc*-*2,* Δ*tul*-*1,* Δ*rho*-*3* and Δ*trs85* mutants to the *clr*-*2* mis-expression strain Δ*clr*-*2* (P*clr*-*2*) [16] and recovered Δ*dsc*-*2;* Δ*clr*-*2* (P*clr*-*2*), Δ*tul*-*1;* Δ*clr*-*2* (P*clr*-*2*), Δ*rho*-*3;* Δ*clr*-*2* (P*clr*-*2*) and Δ*trs85;* Δ*clr*-*2* (P*clr*-*2*) mutants. These strains were then evaluated for their hyper- or hypo-production phenotypes under VMM (sucrose) conditions.

**Fig. 4:**
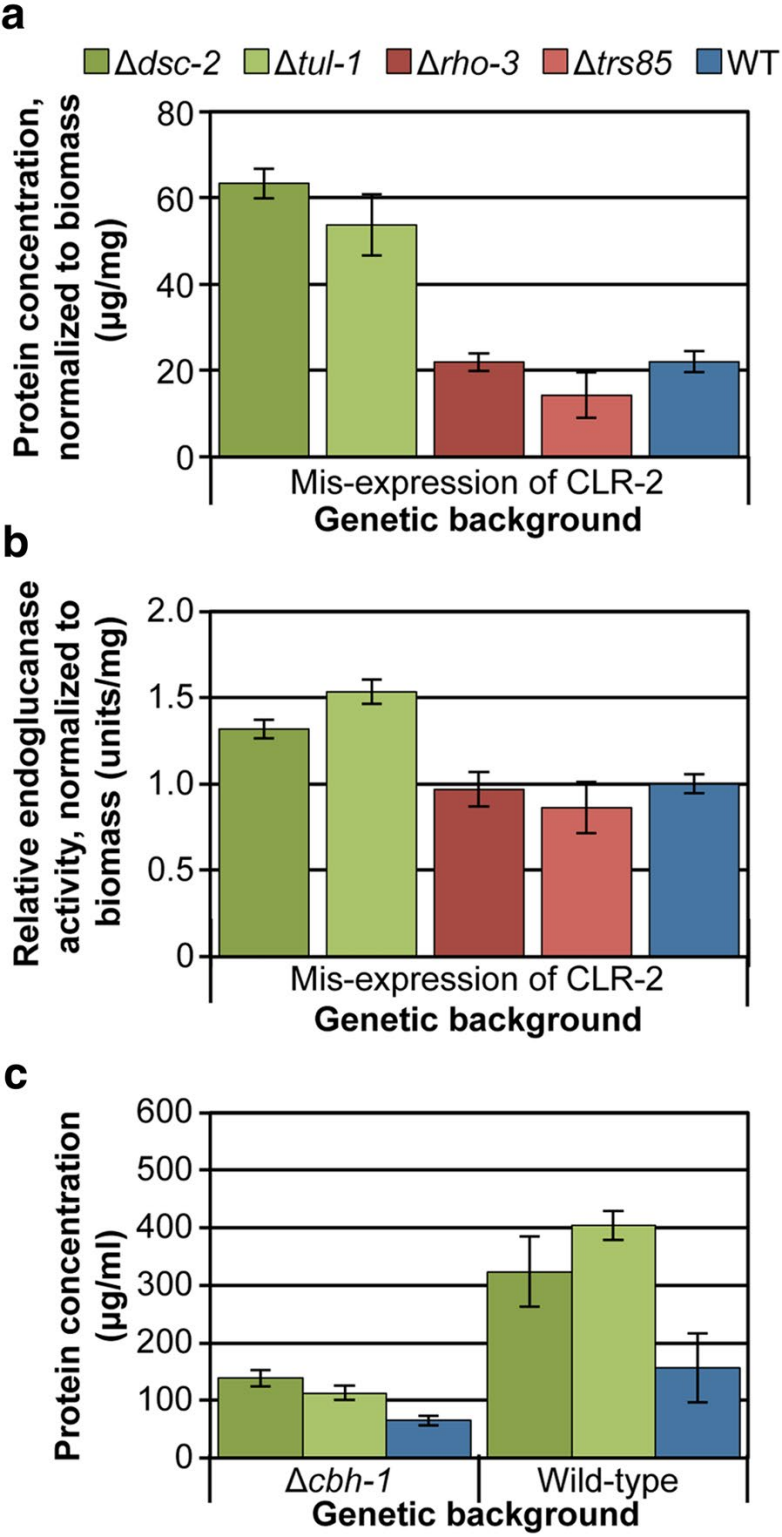
CLR-2-dependence and cellulose and secretory load independence of mutant phenotypes. **a** Total secreted protein and **b** endoglucanase activity normalized for total fungal cell biomass in each culture. Conidia from the indicated strains were cultured in growth media containing sucrose (48 h). **c** Total secreted protein; conidia from the indicated strains were inoculated into VMM (48 h) the resulting biomass then switched to Avicel (48 h). Statistical analyses for protein and enzyme activity levels were relative to WT within each condition (*n* = 3 for CLR-2 mis-expression study; *n* = 4 for Δ*cbh*-*1* study). *Error bars* indicate standard deviation.

The Δ*dsc*-*2;* Δ*clr*-*2* (P*clr*-*2*) and Δ*tul*-*1;* Δ*clr*-*2* (P*clr*-2) strains showed a significant increase in protein secretion as compared to the Δ*clr-2* (P*clr-2*) parental background under sucrose-based VMM conditions and with a corresponding increase in endoglucanase activity (*p* ≤ 0.001 and *p* ≤ 0.01, respectively; Fig. 4), similar to what was observed when the Δ*dsc*-*2* and Δ*tul*-*1* mutants were cultured on Avicel. These data were consistent with the hypothesis that increased protein secretion and enzyme activity was specifically related to the induction and secretion of cellulases as regulated by CLR-2. Unlike the hyper-production mutations, the Δ*trs85;* Δ*clr*-*2* (P*clr*-*2*) and Δ*rho*-*3;* Δ*clr*-*2* (P*clr*-*2*) strains resembled the Δ*clr-2* (P*clr-2*) strain (WT), supporting the hypothesis that the hypo-production phenotype of these mutants is due to a delay in fungal biomass formation in the presence of Avicel, which was alleviated when grown in VMM (Fig. 3). Thus, the hyper-production phenotypes of the Δ*dsc*-*2* and Δ*tul*-*1* mutants were recapitulated by inducer-independent expression of the major cellulase transcriptional regulator, CLR-2.

### The hyper-production phenotype of Δ*dsc-2* and Δ*tul-1* is retained under reduced secretion load

The cellular response of *N. crassa* to the presence of cellulose involves an impressive upregulation in transcription, translation, synthesis and secretion of proteins associated with plant biomass deconstruction [8, 9, 16]. While use of the P*clr*-*2* mis-expression construct allowed for the examination of the Δ*dsc*-*2*, Δ*tul*-*1*, Δ*rho*-*3* and Δ*trs85* secreted protein phenotypes separate from the physical presence of cellulose, these strains still undergo a CLR-2-dependent cellulolytic response, including a large increase in the amount of protein transiting through the secretory pathway [9, 16]. We considered that the hyper-production phenotypes observed in the Δ*dsc*-*2* and Δ*tul*-*1* mutants could be a nonspecific consequence of the increased secretory load that results from CLR-2 induction without being directly dependent on the CLR-2 regulon. If this were true then a high secretory load of CLR-2-independent proteins should also result in the hyper-production of proteins in these mutants. Unfortunately, no other carbon source (including xylan, pectin and BSA) was identified that mimicked the high level of secreted protein observed under cellulosic conditions or when *clr*-*2* is mis-expressed in VMM (data not shown). As an alternative approach, we crossed the Δ*dsc*-*2* and Δ*tul*-*1* mutants to a mutant containing a deletion of the major secreted protein under cellulolytic conditions, cellobiohydrolase-1 (CBH-1) and recovered Δ*dsc*-*2;* Δ*cbh*-*1* and Δ*tul*-*1;* Δ*cbh*-*1* mutants. CBH-1 comprises ~40% of the total Avicel secretome [10] and its absence from the genome significantly reduces the protein load of the secretory apparatus during the cellulolytic response.

The Δ*dsc*-*2;* Δ*cbh*-*1* and Δ*tul*-*1;* Δ*cbh*-*1* mutants were first cultured in VMM in order to generate biomass and then transferred to Avicel medium to induce a cellulolytic response. As previously shown [8], the level of protein secreted in the Δ*cbh*-*1* mutant was approximately half that of the wild-type strain (Fig. 4c). However, the hyper-production phenotype of the Δ*dsc*-*2;* Δ*cbh*-*1* and Δ*tul*-*1;* Δ*cbh*-*1* strains was maintained relative to the Δ*cbh*-*1* parental strain (*p* ≤ 0.001). These data indicated that the hyper-production phenotype of the Δ*dsc*-*2* and Δ*tul*-*1* mutants was not solely due to the secretion load seen in fungal biomass exposed to cellulosic substrates, but was a consequence of induction by cellulosic substrates.

### Identification of the SREBP pathway components that affect secreted protein production under cellulosic conditions

The *N. crassa dsc*-*2* and *tul*-*1* are possible orthologs of *dsc2* and *dsc1* in *S. pombe*, although only moderate sequence conservation was observed (Additional file 3: Figure S2A, B). The Dsc complex in *S. pombe* is composed of five proteins: Dsc1, Dsc2, Dsc3, Dsc4 and Dsc5 [37]. The Dsc proteins form a complex in the Golgi membrane that resembles E3 ligases involved in ER-associated protein degradation [32]. In *Aspergillus fumigatus*, homologs of *dsc1, dsc2, dsc3* and *dsc4* are required for adaption to hypoxic conditions and virulence in a mouse model [38]. The Dsc complex in *S. pombe* and *A. fumigatus* is required for proteolytic cleavage of an ER-membrane bound transcription factor (Sre1 and SreA, respectively) involved in adaptation to hypoxic conditions. The *N. crassa* genome contains predicted proteins with low, but significant similarity to Dsc3 (NCU11245) and Dsc4 (NCU02676) (Additional file 3: Figure S2C, D) and a transcription factor similar to Sre1/SreA (NCU04731) (Additional file 3: Figure S2E). A strain carrying a deletion of NCU04731 was previously characterized in a transcription factor deletion strain set collection and was named for having short aerial hyphae (*sah*-*2*) [5]. We hypothesized that strains carrying mutations in additional components of the predicted Dsc complex and the transcription factor that is the predicted target of this E3 ligase would also show a cellulolytic hyper-production phenotype. To test this hypothesis, single-gene deletion mutant strains for NCU02676 (*dsc*-*4*) and *sah*-*2* (NCU04731) were assessed for their protein production under cellulolytic conditions.

**Fig. 5:**
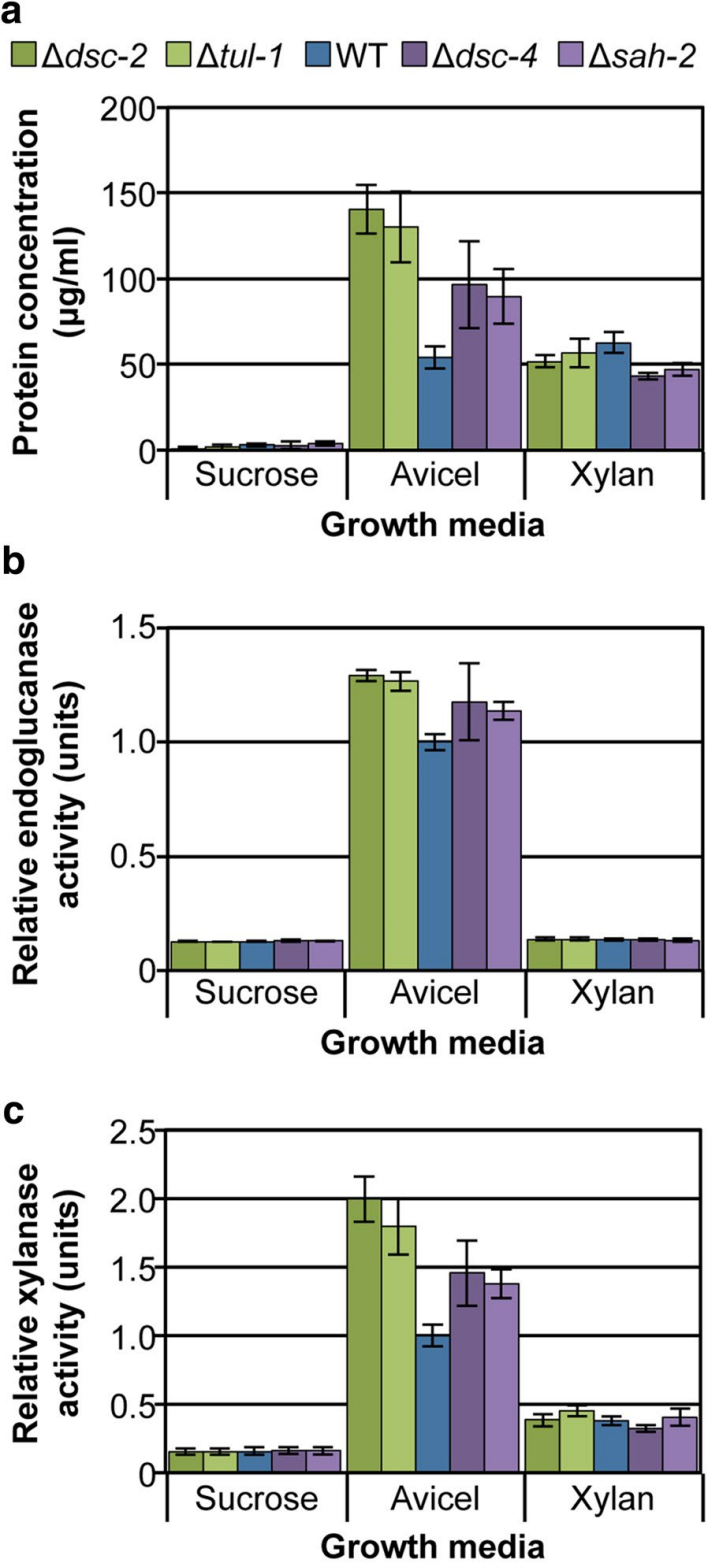
Strains containing mutations in an SREBP homolog, *sah*-*2,* and additional Dsc E3 ligase components show a cellulase hyper-production phenotype. **a** Total secreted protein. **b**, **c** Endoglucanase and xylanase activities, respectively. Conidia from wild type and the deletion strains were inoculated directly into growth media containing sucrose (24 h), Avicel (96 h), or xylan (48 h). Statistical analyses for protein and enzyme activity levels were relative to WT within each condition (*n* = 6). *Error bars* indicate standard deviation.

As observed for the Δ*dsc*-*2* and Δ*tul*-*1* mutants, strains carrying a deletion of Δ*dsc*-*4* or Δ*sah*-*2* showed significantly increased levels of secreted protein and cellulolytic enzyme activity (endoglucanase and xylanase) when cultured on Avicel as compared to the wild-type parental strain (*p* ≤ 0.001 and *p* ≤ 0.01, respectively; Fig. 5). When cultured on sucrose-based VMM, the protein secretion levels of the Δ*dsc*-*4* and Δ*sah*-*2* mutants were identical to the wild-type strain (and the Δ*dsc*-*2* and Δ*tul*-*1* mutants), although under xylan conditions both the Δ*dsc*-*4* and Δ*sah*-*2* mutants secreted slightly less protein than the wild-type strain (Fig. 5a). We subsequently evaluated whether the hyper-production phenotype observed in the Δ*tul*-*1*, Δ*dsc*-*2* and Δ*sah*-*2* mutants was abolished by the introduction of wild-type copies of the *tul*-*1, dsc*-*2* and *sah*-*2* genes driven by the *tef*-*1* promoter. As shown in Additional file 4: Figure S3, the morphological phenotype (short aerial hyphae) of the Δ*tul*-*1*, Δ*dsc*-*2* and Δ*sah*-*2* mutants was fully complemented by the introduction of the wild-type allele of *tul*-*1, dsc*-*2* and *sah*-*2,* respectively. In addition, the protein hyper-production phenotype of the Δ*tul*-*1*, Δ*dsc*-*2* and Δ*sah*-*2* mutants was also abolished by the introduction of the wild-type alleles, as was the corresponding endoglucanase activity (Additional file 4: Figure S3). These data indicate that that SREPB pathway plays an important role in regulating the amount of protein secreted by *N. crassa* under cellulolytic conditions.

Our initial microscopic analyses using the membrane selective dyes FM4-64 and ER-Tracker Red did not show obvious differences in structure between the Δ*dsc*-*2* and Δ*tul*-*1* hyper-production mutants and the wild-type strain (Additional file 2: Figure S1C, D). To further explore this issue, we introduced an ER cellular marker, NCA-1-sGFP or a Golgi marker, VPS52-sGFP [39] into the *Δtul*-*1* and *Δsah*-*2* mutants to generate *Δtul*-*1*; *nca*-*1*-*sGFP*, *Δtul*-*1; vps52*-*sGFP, Δsah*-*2; nca*-*1*-*sGFP* and *Δsah*-*2; vps52*-*sGFP* strains. As with the ER-Tracker Red and FM4-64, no obvious differences in ER or Golgi morphology was observed in the *Δtul*-*1*; *nca*-*1*-*sGFP*, *Δtul*-*1; vps52*-*sGFP, Δsah*-*2; nca*-*1*-*sGFP* and *Δsah*-*2; vps52*-*sGFP* mutants under hyper-production conditions (Avicel) relative to the parental strains (Additional file 2: Figure S1E).

### The cellulase hyper-production phenotype of the SREBP pathway mutants is not simply due to an increased expression level of cellulases

**Fig. 6:**
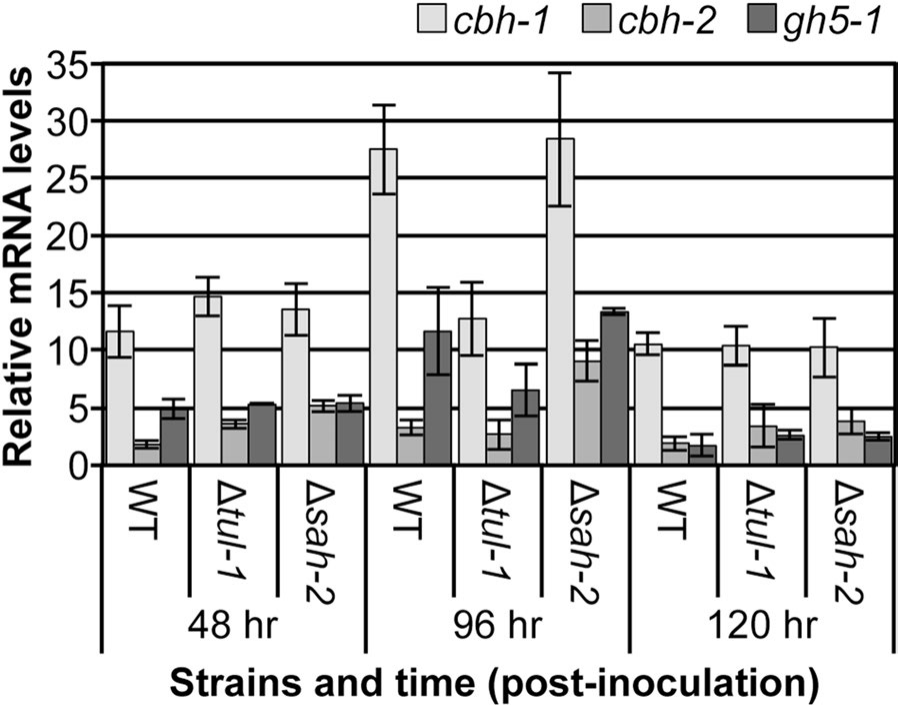
Time course of expression levels of major cellulase genes in the Δ*tul*-*1* and Δ*sah*-*2* mutants relative to the parental wild-type strain under cellulolytic conditions. qRT-PCR experiments measuring gene expression levels from Avicel broth cultures inoculated with conidia from wild type or the Δ*tul*-*1* or Δ*sah*-*2* mutants and grown over a 120 h time course. Relative transcript levels of major cellulase genes *cbh*-*1* (NCU07340), *cbh*-*2* (NCU09680)and *gh5-1* (NCU00762) were normalized to expression levels of actin (NCU04173). Primer sequences used are given in Additional file 5: Table S2. Data from *n* = 3 biological replicates; *error bars* indicate standard deviation.

Our data with the mis-expressed *clr*-*2* strain (Fig. 4) showed that the hyper-production phenotype of the Δ*dsc*-*2* and *Δtul*-*1* strains is related to the CLR-2-dependent regulon. We therefore assessed expression levels of *dsc*-*2* and *tul*-*1* in existing datasets generated for the Δ*clr*-*2* strain when exposed to Avicel relative to the wild-type strain or in strains grown in VMM, but where *clr*-*2* was mis-expressed; neither *dsc*-*2* nor *tul*-*1* showed evidence of transcriptional regulation by CLR-2 [9, 16]. These observations suggested that factors in addition to CLR-2 are important for modulating expression levels of the SREBP pathway components during plant biomass deconstruction. We hypothesized that the hyper-production of cellulases in the SREBP pathway mutants could be due to increased expression of cellulolytic genes in the mutants. We therefore assessed expression levels of the major cellulases (*cbh*-*1, cbh*-*2* and *gh5*-*1*) [10] in *N. crassa* over time in the wild-type strain versus the Δ*tul*-*1* and Δ*sah*-*2* mutants by qRT-PCR. As shown in Fig. 6, the expression of all three of these cellulases was very similar to each other at the 48 and 120 h time point. At the 96 h time point, however, the Δ*tul*-*1* mutant actually showed lower expression levels for all three cellulase genes, especially for *cbh*-*1,* which encodes the major cellulase in *N. crassa* [10] (Fig. 6). Thus, these data do not support the hypothesis that a simple increase in the expression levels of the major cellulase genes in the SREBP pathway mutants is the cause of the hyper-production phenotype.

### Role of Dsc/Sre1 pathway in *Trichoderma reesei*

**Fig. 7:**
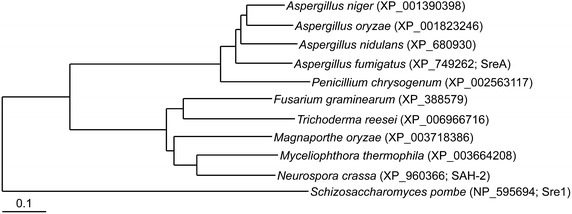
Conservation of Sre1 among industrially relevant fungi and closely related fungal species. Phylogenetic tree of putative Sre1 homologs was generated using MEGA6 [56]. *Scale bar* 0.1 substitutions per amino acid site. *Accession numbers* are noted in the tree.

Single *sah*-*2* homologs were identified across many species of filamentous ascomycete fungi (Fig. 7), suggesting conservation of function. We therefore hypothesized the hyper-production phenotype of the SREBP pathway mutants would not be specific for *N. crassa* and that mutations in these genes in other filamentous fungi that degrade plant biomass would show a similar cellulase hyper-production phenotype. We chose to test this hypothesis in *T. reesei* as hyper-production mutants in this species are used in industrial settings for cellulase enzyme production [40]. A search of the *T. reesei* QM6a genome and its predicted encoded proteins using the *N. crassatul*-*1* and *sah*-*2* protein sequences as queries identified a single ortholog for each. The TUL1 protein in *T. reesei* (Trire2_21342) showed 40% amino acid sequence identity to *N. crassa* TUL-1 (NCU03740) and was predicted as a transmembrane Golgi-localized Ring finger ubiquitin ligase, while SAH2 (Trire2_22774) showed 51% amino acid sequence identity to *N. crassa* SAH-2 (NCU04731) with no annotated functions (Additional file 6: Figure S4). Thus, the *tul1* and *sah2* loci were targeted for deletion in a QM6a strain (Additional file 7: Figure S5).

**Fig. 8:**
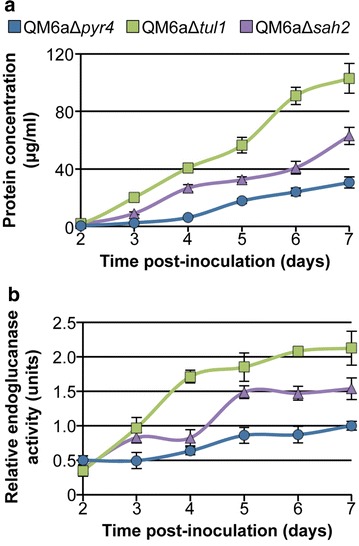
The *T. reesei* *tul1* and *sah2* deletion mutants show a hyper-production phenotype under cellulolytic conditions. Avicel broth cultures were inoculated with conidia and grown for 7 days in shake cultures. Samples taken during the time course were evaluated for **a** total secreted protein and **b** endoglucanase activity. Statistical analysis for protein and enzyme activity levels were relative to the parental QM6aΔ*pyr4* (*n* = 3). *Error bars* indicate standard deviation.

Three independent homokaryotic *T. reesei* Δ*tul1* and Δ*sah2* mutants were selected for morphological and cellulase production analyses. All of the Δ*tul1* and Δ*sah2* mutants showed a similar morphological phenotype to the parental QM6a strain when the only available carbon source was glucose. However, increased hyphal biomass was apparent in the QM6aΔ*tul1* and QM6aΔ*sah2* cultures as compared to the QM6a parental strain when Avicel was used as the single carbon source (data not shown). All three independently derived Δ*tul1* mutants and all three independently derived Δ*sah2* mutants showed increased levels of secreted proteins and endoglucanase activity under Avicel conditions (Additional file 7: Figure S5). The Δ*tul1* and Δ*sah2* mutants displayed higher secreted protein levels and higher enzyme activity during a 7-day time course as compared to the QM6a parental strain (Fig. 8). Similar to what was observed in *N. crassa*, the hyper-production phenotype of the *T. reesei* QM6aΔ*tul1* mutant showed a stronger hyper-production phenotype than that of the QM6aΔ*sah2* mutant (compare Figs. 5, 8 and Additional file 7: Figure S5). Collectively, these data demonstrate that deletion of predicted components of the SREBP pathway results in hyper-production of cellulolytic proteins in two distantly related filamentous fungal species, suggesting that this function is highly conserved among fungi capable of degrading plant biomass.

## Discussion

To identify elements involved in the ability of *N. crassa* to utilize cellulose, we took advantage of a publically available collection of single-gene deletion strains representing nearly every gene in the genome and generated under the auspices of the Neurospora Genome Project [5, 41], a resource currently unique among filamentous fungi. We selected deletion strains based on those genes encoding proteins likely to play a role in cellulase activity or production, including homologs to genes encoding components of the *S. cerevisiae* secretion apparatus, *N. crassa* homologs of genes that showed mutations in the *T. reesei* hyper-production strain RUT-C30, genes encoding the proteins identified in the secretome of *N. crassa* when cultured on plant biomass and genes encoding proteins predicted to traverse the secretory pathway. From this initial set of 567 strains (Additional file 1: Table S1), 25 strains—including seven hyper-production mutants and 18 hypo-production mutants (Table 1)—were identified. Sequence analysis suggested that while some of the deleted genes likely act in transcription, protein synthesis and intracellular trafficking, many of the loci encode fungal-specific proteins of undetermined functions. While the effect of some of these genes/proteins on cellulase secretion is not immediately apparent, for others it is more obvious given their homology to proteins known to impact biomass degradation in other filamentous fungi. For example, the hyper-production ΔNCU07788 (Δ*col*-*26*) mutant was included here because the *T. reesei* RUT-C30 hyper-production strain bears a mutation in *bglR* [22], the ortholog of *col*-*26*. In *T. reesei*, the *bglR* locus is known to play a role in nutrient signaling and cellulase production [26]. It was recently shown that *col*-*26* in *N. crassa* also functions in nutrient signaling and that a Δ*col*-*26* mutant, in conjunction with a deletion of the carbon catabolite repressor, *cre*-*1,* suppresses the inability of a Δ*vib*-*1* (a NDT80-like transcription factor) mutant to utilize cellulose [27].

While each of the identified mutants in Table 1 warrant further characterization, in this study we focused on four deletion strains: Δ*dsc*-*2* (ΔNCU03459) and *Δtul*-*1* (ΔNCU03740), which were included based on their consistent hyper-production phenotype and their predicted role in the secretory pathway, particularly ubiquitination, and the hypo-producers Δ*rho*-*3* (ΔNCU00600) and Δ*trs85* (ΔNCU05213), which are homologous to known elements in *S. cerevisiae* involved in protein trafficking. In the course of our studies, we observed that the hypo-production phenotype of the Δ*rho*-*3* and Δ*trs85* mutants was abolished if first allowed to accumulate biomass in a preferable carbon source (Fig. 3). These results led us to re-evaluate the phenotype of all 18 hypo-production strains identified in Table 1. When first cultivated on sucrose-based VMM before switching to Avicel medium, the majority of these hypo-production strains were found to have similar levels of protein present in the culture supernatant as wild type or a much reduced hypo-production phenotype; this has implications for assessing deletion strain phenotypes in high-throughput screens using conidial suspensions in *N. crassa* and perhaps other filamentous fungi as well. However, three of the identified hypo-production mutants—Δ*ada*-*2* (NCU02017; a transcription factor) [5], Δ*gh3*-*4* (NCU04952; a secreted β-glucosidase) [15] and Δ*stk*-*49* (NCU07062; an uncharacterized protein kinase) [42]—continued to demonstrate significantly reduced protein secretion and cellulolytic activity even when allowed to establish biomass before exposure to cellulose. It is possible that a deletion of Δ*gh3*-*4* may interfere with signaling, since cellobiose (and other cellodextrins) function as inducers for cellulose utilization in *N. crassa* [15]. How the *ada*-*2* deletion strain (which was included in this screen because the *T. reesei* RUT-C30 hyper-production strain also carries a mutation in the *ada*-*2* ortholog [22]) and the *stk*-*49* deletion strain (which was included for its potential role in secretion) impact cellulase secretion remains to be determined.

The hyper-production strains Δ*dsc*-*2* and *Δtul*-*1* showed a consistent increase in protein production and cellulolytic activity in the presence of cellulose and plant biomass as well as when CLR-2 was mis-expressed in sucrose medium (Figs. 1, 3, 4a, b). Although the hyper-production phenotype is linked to the cellulosic response, it is not limited to the production of cellulases (Fig. 2). This effect was not specifically related to an increase in the protein load traversing the secretory pathway under these conditions as an associated increase in secretion is observed even when the overall protein load is reduced, as in a Δ*cbh*-*1* mutant carrying the Δ*dsc*-*2* and *Δtul*-*1* mutations (Fig. 4c). These observations suggest that DSC-2 and TUL-1 play an important role in regulating protein production/secretion under cellulosic conditions, when the organism secretes large amounts of enzymes needed to deconstruct recalcitrant plant biomass substrates.

Subsequent to our initial selection and characterization of the Δ*dsc*-*2* (ΔNCU03459) and *Δtul*-*1* (ΔNCU03740) hyper-production mutants, it was shown in *S. pombe* that homologs to NCU03459 (Dsc2) and NCU03740 (Dsc1) function in a Golgi-localized E3 ligase complex [22]. These two proteins, in combination with three others (Dsc3-5), act upon the transcription factor Sre1 and regulate responses to hypoxia [32, 43]. Sre1 is a homolog of the mammalian SREBP transcription factors [44], which regulate genes encoding enzymes involved in sterol biosynthesis. A number of additional proteins important for SREBP pathway function have been identified and characterized in *S. pombe* [44, 45]. Later studies with the human fungal pathogen *A. fumigatus* showed that homologs to the Dsc complex (DscA-D) and Sre1 (SrbA) also functioned in adaptation to hypoxia, resistance to antifungal drugs and virulence [38, 46]. More recently, it was shown that a direct target of *A. fumigatus* SrbA was another transcription factor, SrbB, which has been implicated in the regulation of carbohydrate metabolism [47]. In mammalian cells, loss of SREBP caused changes in lipid composition and induced accumulation of reactive oxygen species, cell death (apoptosis) and a reduction in protein synthesis as a result of ER stress [48]. The linkage we identified between the loss of the SREBP pathway, which was not previously known to be involved in protein secretion, and the hyper-production of cellulases demonstrates the strength of screening deletion strain collections in an unbiased manner. Future studies in *N. crassa* will dissect the relationship between regulation of secretion by SAH-2, the Dsc E3 ligase complex and accessory proteins under cellulolytic and plant biomass conditions versus the role of this pathway under hypoxic conditions.

Although homologs of SAH-2 are well conserved among filamentous fungi (Fig. 7), it was possible that the hyper-production phenotype associated with mutations in the SREBP pathway components would be restricted to *N. crassa*. To test this hypothesis, we constructed strains carrying deletions of *dsc1* and *sah2* in the *T. reesei* strain QM6a. Neither of these mutations occur in the hyper-production RUT-C30 strain [22, 23]. The QM6aΔ*dsc1* and QM6aΔ*sah2* strains secreted significantly higher quantities of enzymatically active cellulases into the culture medium as compared to their parental strain (Fig. 8, Additional file 7: Figure S5). These data suggest that the role of the Dsc/SREBP pathway in regulating secretion of enzymes needed for plant biomass degradation is conserved between two distantly related filamentous fungi, *N. crassa* and *T. reesei*. Better understanding and modification of the components of this pathway could allow for further engineering of increased protein production in both these species as well as other industrially relevant filamentous fungi.

## Conclusions

In screening a panel of *N. crassa* single-gene deletion mutants, we identified an association between the hyper-production of secreted cellulases and components of the SREBP pathway that is apparent only under cellulosic conditions. Previous studies on mutants in this pathway had not linked it to a role in protein secretion. Importantly, the hyper-production phenotype that accompanies the deletion of DSC and SAH-2/SRE-1 is conserved between distantly related species of filamentous fungi. The continuing characterization of *sah*-*2*, the *dsc* E3 ligase and accessory protein mutants will enhance our understanding of the ability of *N. crassa* to utilize complex carbon sources present in its natural environment, while broadening our knowledge of protein secretion in filamentous fungi, including industrially relevant species.

## Methods

### Neurospora strains and growth conditions

Single-gene deletion strains were obtained from the Fungal Genetics Stock Center [5, 6]. The Δ*clr*-*2* (P*clr*-*2*) mis-expression strain [16] was used for crosses to construct double mutants carrying the Δ*clr*-*2* (P*clr*-*2*) and Δ*dsc*-*2,* Δ*tul*-*1,* Δ*rho*-*3* or Δ*trs85* mutation, which were verified by phenotypic screening and PCR. The *Δtul*-*1*; *nca*-*1*-*sGFP* and Δ*tul*-*1; vps52*-*sGFP* strains were constructed by transforming a *his*-*3*; Δ*tul*-*1* strain with the plasmids *nca*-*1*-*sGFP* and *vps52*-*sGFP* [39], respectively. The Δ*sah*-*2; nca*-*1*-*sGFP* and Δ*sah*-*2; vps52*-*sGFP* strains were created by crossing strains bearing NCA-1-sGFP or VPS52-sGFP into the *Δsah*-*2* mutant. Genotypes were verified by phenotypic screening and PCR. Complementation was performed by inserting the *tul*-*1, dsc*-*2* or *sah*-*2* gene into the Ptef1-Tcyc1 plasmid and transforming it into the *csr*-*1* locus by selection for cyclosporin resistance [49]. Genotypes were verified by phenotypic screening and PCR.

Liquid growth media comprised 1X Vogel’s salts in ddH_2_O plus 2% weight-per-volume carbon source, while all solid media contained an additional 1.5% agar. Carbon sources included: sucrose, Avicel PH-101 (Sigma 11365), xylan (from birchwood, Sigma X0502 or beechwood, Sigma X4252), sodium carboxymethyl cellulose (CMC; Sigma 419273) or ground *Miscanthus x giganteus* (0.25 mm diameter). *N. crassa* strains were inoculated from freezer stocks into slant tubes of sucrose-based Vogel’s minimal medium (VMM) agar [25] and incubated at 30°C in the dark for 2 days followed by at least 4 days incubation at 25°C in the light. Conidia were harvested in sterile water and then inoculated at 10^6^ per milliliter into 3 mL of growth medium aliquoted into 10-mL deep-well, square-side, round-bottom 24-well plates (GE Healthcare 7701–5102) or 100 mL growth medium in 250-mL Erlenmeyer flasks. Plates were covered with a breathable, sterile sealing tape while flasks were capped with a sterile foam stopper; both were then incubated at 25°C in constant light with rotation (200 rpm) for 24–96 h.

For switch experiments, conidia were inoculated into 3 mL VMM and cultured for 48 h as above. The fungal biomass was then washed with a solution of 1X Vogel’s salts in ddH_2_O before transferring to 3 mL of freshly aliquoted growth medium. The cultures were then incubated at 25°C in constant light with rotation (200 rpm) for 48 h.

For all experiments, *N. crassa* strains were cultured in replicates of at least three for statistical analysis by one-way ANOVA using the Dunnett’s multiple comparisons test.

### Construction of *Trichoderma* mutant strains and growth conditions

A wild-type strain of *T. reesei*, QM6a (ATCC 13631), was obtained from the American Type Culture Collection. An uridine auxotroph strain, QM6aΔ*pyr4*, was generated via the targeted deletion of *pyr4* and used as the recipient for generating the *tul1* and *sah2* deletion mutants, QM6aΔ*tul1* and QM6aΔ*sah2*. The *pyr4* deletion vector, pSC*pyr4*, was constructed by ligation of 5′ flanking DNA, the *hph* marker gene for hygromycin resistance, and 3′ flanking DNA into a vector backbone. The sequences of primers used in the construction of the *T. reesei* deletion vectors are provided in Additional file 5: Table S2. The following fragments were amplified from QM6a genomic DNA: the 5′ flank of *pyr4* (2.1 kb) was amplified using primers pSC-pyr4A-F and pSC-pyr4A-R, whereas the 3′ flank of *pyr4* (1.9 kb) was amplified using primers pSC-pyr4C-F and pSC-pyr4C-R. A hygromycin-resistance cassette (2.3 kb) including *hph* was amplified from the genomic DNA of the *N. crassa* Δ*dsc*-*1* strain using primers pSC-pyr4B-F and pSC-pyr4B-R. The backbone of the vector (3 kb) was amplified from the plasmid pMF272 [50] using primers pSC-pyr4D-F and pSC-pyr4D-R.

For deletion of *pyr4*, the split marker technique was used to improve homologous recombination [51]. Split-marker fragments of the *pyr4* deletion cassette were amplified from pSC*pyr4* by PCR using the primer pairs pSC-pyrA-F with pSC-SM-R and pSC-SM-F with pSC-pyr4C-R. Strain QM6a was transformed as described [52]. Transformants were screened on glucose-based minimal medium (MM) agar [53] supplemented with hygromycin and deletion of *pyr4* was verified by PCR (Additional file 5: Table S2).

To generate the QM6aΔ*tul1* and QM6aΔ*sah2* deletion strains, a *tul1* deletion vector, pSC*tul1*, was constructed by ligation of 5′ flanking DNA, the *pyr4* marker gene, 3′ flanking DNA and a vector backbone. The following fragments were amplified from QM6a genomic DNA: sequence 5′ to *tul1* (2.4 kb) was amplified using primers pSC-tul1A-F and pSC-tul1A-R; the *pyr4* cassette (1.8 kb) was amplified using primers pSC-tul1B-F and pSC-tul1B-R; and sequence 3′ to *tul1* (2.5 kb) was amplified using primers pSC-tul1C-F and pSC-tul1C-R. The backbone of the vector (3 kb) was amplified from the plasmid pMF272 using primers pSC-tul1D-F and pSC-tul1D-R. Split-marker fragments of the *tul1* deletion cassette were amplified from pSC*tul1* by PCR using the primer pairs pSC-tul1A-F with pSC-SM-R and pSC-SM-F with pSC-tul1C-R. The *sah2* deletion vector, pSC*sah2*, was constructed in a manner similar to pSC*tul1*. Three fragments were amplified from QM6a genomic DNA: sequence 5′ to *sah2* (2.0 kb) was amplified using primers pSC-sah2A-F and pSC-sah2A-R; the *pyr4* cassette (1.8 kb) was amplified using primers pSC-sah2B-F and pSC-sah2B-R; and sequence 3′ to *sah2* (2.5 kb) was amplified using primers pSC-sah2C-F and pSC-sah2C-R. The backbone of the vector (3 kb) was amplified from the plasmid pMF272 using primers pSC-sah2D-F and pSC-sah2D-R. Split-marker fragments of the *sah2* deletion cassette were amplified from pSC*sah2* by PCR using the primer pairs pSC-sah2A-F with pSC-SM-R and pSC-SM-F with pSC-sah2C-R. The split-marker fragments of the *tul1* and *sah2* deletion cassettes were transformed into QM6aΔ*pyr4* as described [52]. Transformants were screened on MM agar and the deletion *tul1* and *sah2* was verified by PCR (Additional file 5: Table S2, Additional file 7: Figure S5).

*Trichoderma reesei* strains were inoculated onto potato dextrose agar (PDA) plates, supplemented with 5 mM uridine or 300 μM hygromycin when necessary, and incubated at 30°C for 5–7 days. Conidia were harvested using a solution of 0.9% sodium chloride and 0.1% Tween 80 and then inoculated at 4 × 10^6^ per milliliter into 50 mL MM supplemented with 1% Avicel prepared in 250-mL Erlenmeyer flasks. The flasks were incubated at 28°C with rotation (200 rpm). Samples of the culture supernatant (1.5 mL) were taken for protein level and enzyme activity assays every 24 h. *T. reesei* strains were cultured in biological replicates of at least three for statistical analysis by one-way ANOVA using the Dunnett’s multiple comparisons test.

### Protein and enzyme assays

Culture supernatants were harvested from the *N. crassa* and *T. reesei* cultures; when necessary, particulate matter was removed by flushing the supernatants through a 0.2-μm filter. Total secreted protein levels were determined by Bradford assay (Bio-Rad Protein Assay). The Azo-CM-Cellulose reagent (from Megazyme) was used to determine endo-1,4-β-glucanase activity levels; endo-1,4-β-xylanase activity levels were determined using Azo-Xylan (Birchwood) (also from Megazyme). Avicelase assays measuring the release of glucose and cellobiose from Avicel substrate were performed as in [8]. All enzyme activity assays were scaled down in volume to be run in 96-well or deep-well 96-well plates.

### Microscopy

For microscopy studies, conidia were inoculated onto agar plates or liquid Vogel’s with 2% Avicel and then incubated overnight at room temperature. Microscopy studies were performed using a Leica MZ 16F stereomicroscope or a Leica SD6000 spinning disk confocal microscope with a 488 or 561 nm laser. Fluorescent dyes included FM^®^ 4–64 for staining plasma and vesicular membranes and ER-Tracker™ Red for staining the ER (both from Molecular Probes).

### SDS-PAGE and immunoblots

Protein samples were combined with Laemmli sample buffer and then loaded on 10% Criterion™ Tris–HCl Precast Gels (from Bio-Rad) and run at a constant voltage. Coomassie staining was achieved using GelCode^®^ Blue Stain Reagent (from Thermo Scientific). For immunoblot, proteins were transferred to nitrocellulose membrane using a Trans-Blot^®^ SD Semi-Dry Transfer Cell system (from Bio-Rad). Specific proteins were detected using peptide antibodies generated by Thermo Scientific’s Pierce Custom Antibody Services: CDH-1 (NCU00206), GH5-1 (NCU00762), GH10-1 (NCU05924), GH61-5 (NCU08760), GH51-1 (NCU02343) and GH10-2 (NCU08189). In addition, an antibody directed against GLA-1 (NCU01517) was kindly provided by Stephen Free. These primary antibodies were detected using a goat anti-rabbit IgG (whole molecule)-peroxidase antibody (from Sigma) secondary antibody. The specific protein bands were visualized using the LumiSensor™ Chemiluminescent HRP Substrate Kit (from GenScript) and ChemiDoc™ XRS+ molecular imager with Image Lab™ software (from Bio-Rad).

### Sequence analysis

When possible, protein sequences were obtained from either the *N. crassa* genome database maintained by the Broad Institute (http://www.broadinstitute.org/annotation/genome/neurospora/MultiHome.html); the *T. reesei* genome database (http://genome.jgi-psf.org/Trire2/Trire2.home.html); the *S. cerevisiae* genome database (http://www.yeastgenome.org); the *S. pombe* genome database (http://www.pombase.org); or the *A. fumigatus* genome database (http://www.aspergillusgenome.org). Sequences from other select fungal species were identified using the BlastP algorithm [54], which was also used to evaluate similarity between two sequences. Alignments of multiple protein sequences were performed using MUSCLE [55]. Evolutionary relationships between multiple protein sequences were analyzed with MEGA6 [56]. The phylogenetic tree for putative SAH-2 homologs was built using the neighbor-joining method: evolutionary distances were computed using Poisson correction and all gaps were eliminated from the data set.

### Quantitative RT-PCR analysis

An aliquot of 2 × 10^6^/mL of *N. crassa* conidial suspension was directly inoculated into 100 mL liquid Vogel’s with 2% Avicel as carbon source in 250-mL Erlenmeyer flasks and cultured at 25°C in constant light with rotation (200 rpm) for 120 h. Biological triplicates were prepared for each strain. Total RNA was isolated from samples taken at 48, 96 and 120 h post-inoculation using zirconia/silica beads and a Mini-Beadbeater with 1 mL TRIzol reagent (from Invitrogen). RNA purification and DNase I treatment were performed using RNeasy Mini Kit (from QIAGEN). qRT-PCR was performed using EXPRESS One-Step SYBR^®^ GreenER™ Kit (from Invitrogen) on the CFX Connect™ Real-Time PCR Detection System (from Bio-Rad). Primer sequences used for amplification are described in Additional file 5: Table S2. The relative transcript level of major cellulase genes *cbh*-*1* (NCU07340), *cbh*-*2* (NCU09680), and *gh5*-*1* (NCU00762) were normalized to expression from the actin gene (NCU04173) and calculated by $2^{{ - \Delta C_{\text{t}} }}$ as a relative expression level.

## Additional files

**Additional file 1:** **Table S1.** Single-gene deletion strains available from the Fungal Genetics Stock Center that were included in screen.
**Additional file 2:** **Figure S1.** Phenotypic characterization of hypo- and hyper-production strains.
**Additional file 3:** **Figure S2.** Dsc protein complex sequence alignments.
**Additional file 4:** **Figure S3.** Deletion of Dsc E3 ligase complex and SREBP1 component homologs showed a short aerial hyphae and resulted in higher cellulase production.
**Additional file 5:** **Table S2.** Primers used in this study.
**Additional file 6:** **Figure S4.** DSC-1 and SAH-2 alignments with *T. reesei* protein sequences.
**Additional file 7:** **Figure S5.** Generation of *tul1* and *sah2* deletion mutants in *T. reesei* and their corresponding hyper-production phenotypes.

## Abbreviations

CBH: Cellobiohydrolase
CLR: Cellulose degradation regulator
DSC: Defective for SREBP cleavage
GH: Glycosyl hydrolase
SREBP: Sterol regulatory element binding protein
VMM: Vogel’s minimal medium
MM: Minimal medium
