# Supplementary material for: Deletion of homologs of the SREPB pathway results in hyper-production of cellulases in Neurospora crassa and Trichoderma reesei
Source: Biotechnol Biofuels. 2015 Aug 19;8:121. doi: 10.1186/s13068-015-0297-9 (PMC4539670; doi:10.1186/s13068-015-0297-9)
Supplement: Supplementary file 2 — Additional file 2: Figure S1. Phenotypic characterization of hypo- and hyper-production strains. [file 13068_2015_297_MOESM2_ESM.pdf]

Figure S1

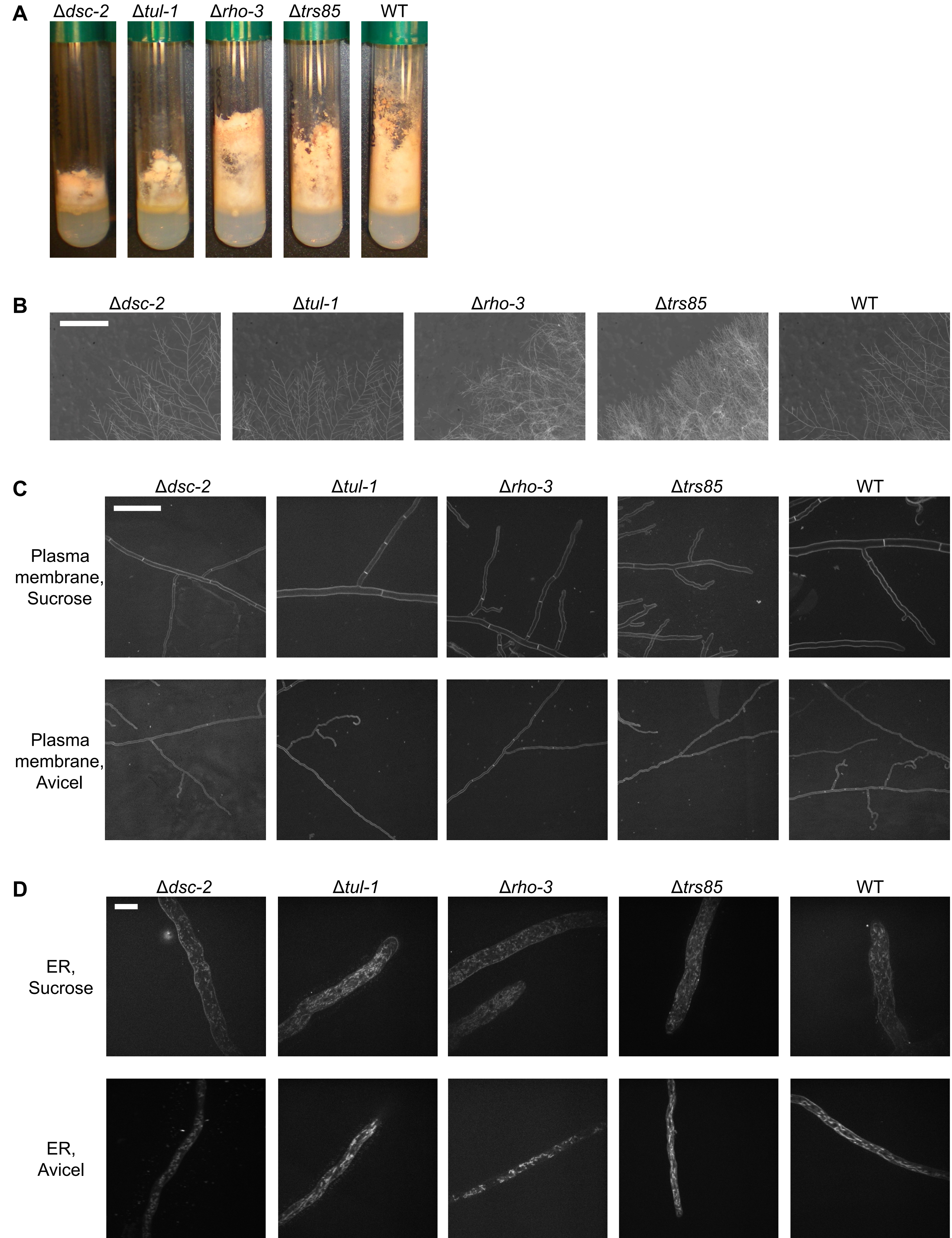
A
Δdsc-2
Δtul-1
Δrho-3
Δtrs85
WT
B
Δdsc-2
Δtul-1
Δrho-3
Δtrs85
WT
C
Δdsc-2
Δtul-1
Δrho-3
Δtrs85
WT
Plasma membrane, Sucrose
Plasma membrane, Avicel
D
Δdsc-2
Δtul-1
Δrho-3
Δtrs85
WT
ER, Sucrose
ER, Avicel

E

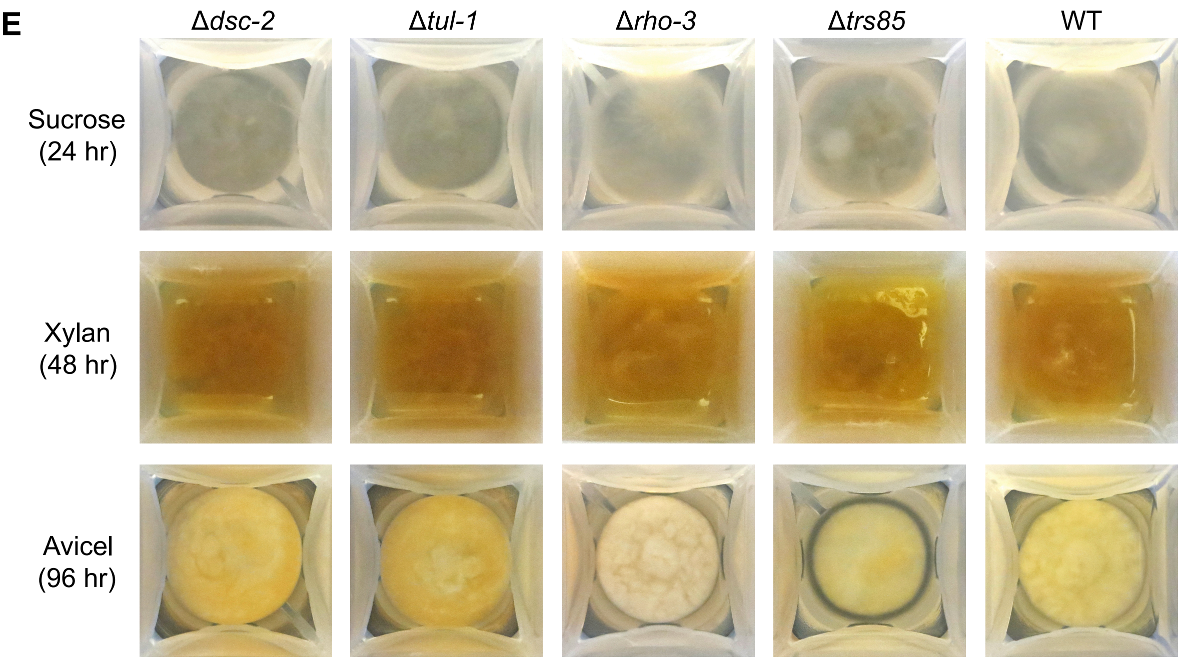

F

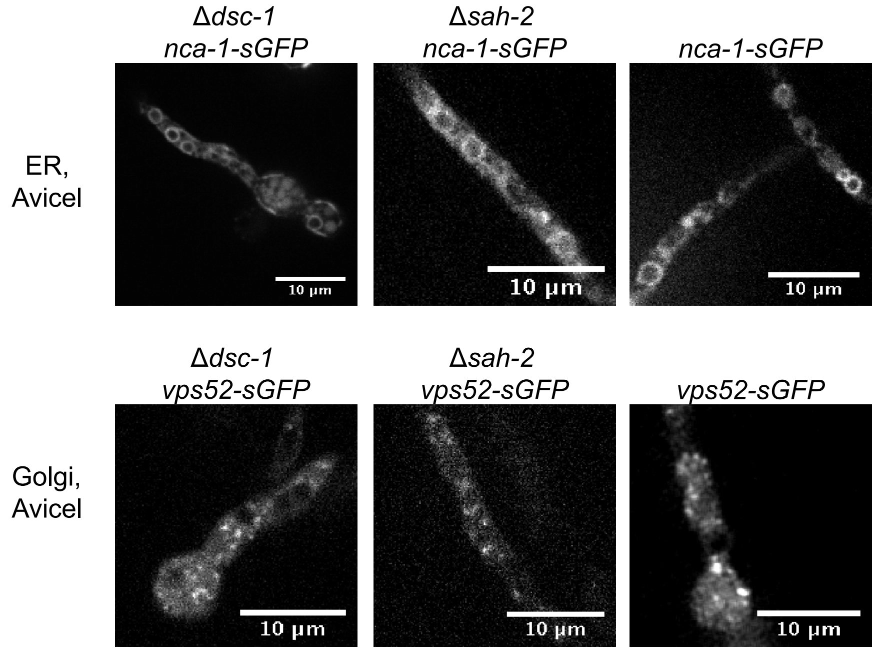

Figure S1. Phenotypic characterization of hypo- and hyper-production strains. (A) Gross morphology on VMM Agar. **(B)** Bright field microscopy of morphology at leading edge of colonies on VMM Agar. Scale bar = 1mm. **(C)** Fluorescent microscopy of cellular morphology from colonies on VMM or Avicel agar using plasma membrane (FM4-64) stain. Scale bar = 100 μm. **(D)** Fluorescent microscopy of cellular morphology from colonies on VMM or Avicel agar using ER (ER-Tracker Red) stain. Scale bar = 10 μm. **(E)** Growth in VMM, xylan and Avicel broth. **(F)** Fluorescent microscopy of cellular morphology from germlings containing sGFP-tagged NCA-1 (ER marker) or VPS52 (Golgi marker) on Avicel agar. Scale bars = 10 μm.
