# Supplementary material for: Deletion of homologs of the SREPB pathway results in hyper-production of cellulases in Neurospora crassa and Trichoderma reesei
Source: Biotechnol Biofuels. 2015 Aug 19;8:121. doi: 10.1186/s13068-015-0297-9 (PMC4539670; doi:10.1186/s13068-015-0297-9)
Supplement: Supplementary file 3 — Additional file 3: Figure S2. Dsc protein complex sequence alignments. [file 13068_2015_297_MOESM3_ESM.pdf]

Figure S2

A

```
Dsc1      MDRRRWVPSTPVVTLLLLFMLFAPAPRLP---SRNGESSEKSIKAEKRAFSEIKNATFLNIPE---------------------------
DscA      MDNRGS----FFFLLIVFYLLLSSQSRPP--LLDQDRERQREVARERDALRLLNESKYGDFDP---------------------------
NCU03740  MPSQNSDVRALLIVLILLWIFFSPDSSDPASLTPPEIAHARE-ARFQGALDVLNTTRWGDFAPDAPSSTSPRPPSETDGPAGEIGKGNGD
          *   .         .. *::::::::. .   *             ..     . *:   :: : : ::

Dsc1      ---RVEHSLTFP--------TEIWE------------TRDGL----------FEEPVGKGD---------SHLNHTSVMTGNWN------
DscA      ---PGDKWLPFAGTRKND--SYAWGILPEAQGRARHQLRSAISNAGLEPPRSLEDPDALPSLNLTQLLLPVYRNATGKLRGDWVRR--KL
NCU03740  KHEQGPQFLNITGFSQLDGKGYAWEDLGRFRNRCREWSRNAFPSAATVGP--LDDDSWEPS-----MVSGTWKNATGTLHGTWVRRPGTV
                : * :.          *             *..:           :::      .              * *. : * *

Dsc1      ILPYPSFG--KVSPNVTWHTTLRNI---VMSQSGRFSANL---------YEYVD-------GNSDGISFVLNLENNN-DTSVYHMTFHGD
DscA      NKEYPKLNTTAIALEHGYFTH--EFGLNITGSSGTFYLDLREGGGE---ELRVD------SGQVREIRATLAVESNDFWGNTWYISLFGV
NCU03740  VRQAAEYNLSAIAPGATWPSSLGEWGRNITGDHGKIELRIDEDEGDGVYEEKVEGKTSRGANLAREIQAVATIQDDASEASSWSLLLHGV
              .. .    ::     : :    :     : .. * :    :              *:         .     *   .   ::.:     . : : :.*

Dsc1      RVKPINVFLGSTDVTPNFGGVDVIPWLLKDSPYKDAPPLDGTEYFPLLQNRSLERIETRLQDAQT-------VGWSPLVFEEE----EVT
DscA      HFPETGAIILSSN-SEKFEGLFVLPHL--------AF---SSDAYELSHQLLLNSLSDTLSEKENRPPTL--FPWSSLIGSEQVEFPAPK
NCU03740  HWPRQGAILLTTS-SEKFDGIFGLPHL--------AP---GPNFFHTGQHLLNETLGKIIQARKKSKYGFPGNPWGSRMEDDD-SFPA-Q
          .    ..:: ::. : :* *:  :*.*           *     ..: :    ::    : :    :.   :.           *.. :  .::

Dsc1      CSAFVFLHNKNTGLDKET---------LKAIENEFYHPQGVSTQKMPEVFVSGLVYSPDCNVAFTFSNTKGPRNF--------------V
DscA      CEHIIYLQQHPVTI-HDYLADKPV---VDQIEEELRFPIGAPVPPAPLMVMSAVVFSPDCGY---ILETKGTPDFPPSEGLYLTGPKIEE
NCU03740  CEYVMYLQLQPLELSRQYESPQLLESYIEELERELRHPTGAPIGRIPDLRASMVAWSPDCSF---FLESKGPPEFPSVDGEHLVGMKEAM
          *. .::*: :    :  .:            :. :* *: .* *..     * :   * :.:****.     : ::**. :*

Dsc1      LENHLVRFSSLYIFIVLSQIFVLLRQMRI-NSPSHVQRLSFLTIAMQAGLDAYIAIF--FLSTNAVIEKGYLPFVSVAFLSLVPSVMFTM
DscA      YDKYSARLVFIICGVFAAQITLLLRQIKEASTPSTRSRISFYTIALMAFGDAFVLIF-ILLELYPAVS--FLVMATAAFLTFLSVSYIGM
NCU03740  FTYTAKEWLIAFAAVMLGQIWLFKEQMKLSNTPSTTGRVSFWTIGAMLFADGMIFVTACAWSLNATIT--LLPCLLVTFAAFIS-MFIEG
                         :. .** ::   *:.   .:**    *:** **.       *. : :      .   ..:     *      .:* :::.    :

Dsc1      RYLALILRVQ-----------------------------NSNMPPPAPRPVTNNSSNNNTNQSNASNENSPNAPSAANDNTE--------
DscA      KFMMEIWAVQAPERREQER--------------------RSNPPASTPRS-TGLPLPATSAPVRDSGAT----PIILTPDQD--------
NCU03740  AFLSEVWKIQEPERRIREREREQAAAAAAAAAAANRNASSSSAASTTPPP-TTQPEPPTTQPAPPPPPSQPRQPIIVPSDGDIDEAEQLN
           ::   :   :*                                 *.  ...:* . *   .     .:       .    .      *      : :

Dsc1      -----TTTVNPPQEDDQP----MTQHERDQR-DWSAVCLRFYFIILVVCIASLYSAFWPVIYRFYFISALIFTSYSFWIPQIIQNVKQGT
DscA      ---------PPAEEDDQPTNRGTTSAAQETRNDVGAMYARFYFVLFVMLIISIWSFLWPNRLGALYARALAFVYLSFWTPQIGRNIIRNC
NCU03740  NLLSGASALPLPATARQPGLPAPTNEPRPPT-PFSTITGRFVLAGLFLLFISAVATSWPSHIRSFYVNTLSFLYLSLWTPQIIRNAQRNS
                         .     **       *.   .         .::   ** :   :.: : *    :   **          :   :* *     *:* *** .*    ..

Dsc1      SRSFTWTYILGASVLRLYLPLAIFIDSELILGFPPKYFFALGLVLWMLFQVLVLLVQDTLGPRFFLPKKFFLSSPVYDYHPVIQQ-----
DscA      RKALRWDFVIGQSILRLFPFVYFLTVRGNVLFIHPDTTTAFALAGWVWIQVWVLASQDILGPRFFVPRGW--APAAYDYHPILRD-----
NCU03740  RQAFSWRFMIGQSLLRLLPFAYFFLREDNVLLADPDPFAFLVLVAWVWIQLWVICAQSILGPRFGVPKGW--VREAWDYHPVLRDDDLEG
           .:: * :::* *:***      ::      :*    *.     : *. *: :*: *:    *. ***** :*. :       .:****::.:

Dsc1      ------------------------------------------------------------DDLEAFMRDANV------------------
DscA      ---------------------------------------GDESEADLESGGVLPIGALRAEDLSGDAKDEDKQRTKDRKRA------VFD
NCU03740  VSLPIGLARTGTGLSSSSSSSSSPTEERVRGRAWSVVSLGGPAAAAETGEGSAAAAAAAAATDSAGGTRNREREKEKEREKQKGVTMRSVD
                                                                                                 *   .   .: :

Dsc1      CPICMQPIELV-------STGS----TLNPASMMVRRNYMLTPCHHLYHRQCLLQWMETRSICPVCRCHLPAV
DscA      CAICMQEIEVPVLAARGSAGGS--SVTEGATSILSRRTYMVTPCRHIFHSTCLESWMRLRLQCPICRESIPPV
NCU03740  CAICRELLEVPVFNNNKGSGGSSDSAASGITGVFARKAYMVTPCRHIFHTNCLEGWMKYRLQCPICREELPPL
          *.** : :*:          : **     : . :.:: *. **:***.*::*  **   **  *   **:**   :*.:
```

B

```
Dsc2        MSSANIVPSNMGITKFLLLTISTSSVVAGVFALKPFFHINFGLHLLSHYQYWRILLWQFI-YWNSTEVFQALFIIYQARDVERLLGSHRF
DscB        MLTSGL--TNAPITKLLLIYTIASSIALSILDIKHLASIHVSPHLWPYAQFWRLATWQLAGFTNSTEALFAAMLAYHLRVVERAWGKRKF
NCU03459    ---MSF--TDAPVTKALVLGLVGLSIAASVFDIKHYFYISIGTHFLRYGQLWRMLTYQLC-YTNSSEVLFGAMSLYHMRMVERFWGSRKY
               .:  ::  :** *::     *:. .:: :*    * .. *:  : * **:  :*:  : **:*.: . :  *: * ***  *...:

Dsc2        ASFCVYMFILGMFVTPIF--SFLYSLLFKNLDYIQPGPTFLIFAILYQYYYIVPSTVFVRLFN---------------------------
DscB        ATFIISTLPYTSLLPPLLL-VLLRPLTLYKLNYLPCGPTATLFALLAQYHAGIPHTFRYRISTSTSTSTSSANRDTDASGRGEGAQGKHL
NCU03459    ASFILLAALFTAIIPPIFLTVVLRPLSFGVLDYIPAGPTPILFAILAQYHAMIPQIYKYKVALSAKTPSNASD---DTSG----------
            *:* :       ::.*::   .* .* :   *:*:  ***  :**:* **:  :*     .:

Dsc2        -IKFTDKFQMVIPMIGLAFSHFPSTFINAFLGWTMGMFYHLSLLPGTS--WRLPIRFVKPALSPTHVFIRPPYSDMQNASTFNPETLFAL
DscB        TLLLSDKSTTYLVAAQLALSQFPGMMLPAAVGWVVGVAWRAEVLPVPSARWRVPAWAVGEKEMGRR----------------------GT
NCU03459    -FIFSDKSTRYLMALQLALFQWPGSLLGAVIGWAVGYLWRNDLLPSAVARWRVPGWVV----------------------------GL
             : ::**    :    **: ::*. :: * :**.:*  :. .:** .   **:* . *                          .

Dsc2        PTGLDAERTENENQVENPVSNADANDSPTRQNARATAIASSSNTAASFRNRQQISHPPLGRTSSSSVLPTGPASQLYDMLSGRSERPELG
DscB        QGGEGGERYED-----------------LRRRLEGEAVAAAAAASGN----------------------AGSASE----ASGQRQRRREG
NCU03459    RAQKRNDRFDG-----------------IRRRLEDEGASTGVATGA-----------------------QPAAE----GEGSRRRPLGQ
                  :* :.                 *..    . ::.  :..                       .*::     .*   *

Dsc2        NIREEDINTVQTIMQTSRAQAIQALSQTNDVQRAVELLLEQTADY
DscB        GIMDR----------------LRAL--------------------
NCU03459    QLIDE----------------VRGAF-------------------
             : :                 :..
```

C

```
Dsc3        ------MSSSALK----KWE-----------------IVIRFASSIPDLSLEISDAQTTTIHSLFKIVRNRIPE-CRD-----------K
DscC        ------MTFPAFLSPDPTWDGDSTGG--------PLLLTVRFSASIPDFPLDIENPDITTAAGLKQLIRTHLPPNLSS-----------H
NCU11245    MSDDWENSSPSNRASRPPVSRVNTASSPNKPFSPSLHLTVRFSASLPDLHLDIPSPNRTTIVALKHRIRERLLSSSKDSKDETTVKAARS
                   : .:        .                 :.:**::*:**: *:* ..: **  .* : :* .:      .

Dsc3        QLKMVFQGRLLSPGFTVERA-------------------VRGNWQRDENDDPNIVQKAFIHCIVGP-TLTEEELASQDQAQSGLNSNSES
DscC        RLRLIYAGRGLEDATPLSVSLKLPPSPSRTPVVQEDATTVKGKGKAPIREQP----RLYIHCSIGDIVLSDADLAAEAAIATTLQQEQAD
NCU11245    RLRFIHAGKILPDSAIVSSVLRPPPPPPMIMD-------TKGKGKAVDRQQQQQQQRVYVNCSIGD-ELTDEELATEAAAAAFVPVPVGS
            .*.::. *. *  .  :.                     ..*: :   .::      . :::* :*   *:: :**::     : :     .

Dsc3        PDD-------------------LQNAQTGETL---------------------------RGFDRLREAGFTETEVNNLRSQFHRLRGTNL-
DscC        EDY-------------TGRKKQQQPPPSTTSA--------------------------PRGFDRLLSAGFTPSEVSALRSQFMAIQSVSRT
NCU11245    VDSNLSGTGSGAGGGKTSRTTGLQPSSSSSSTGESRSRINASAGQATTTTTTTDRNHPRGFDRFLSAGFTPAEVNQLRLQFTSIQASRFT
             *                    * . :  :                          *****: .**** :**. ** **  :..

Dsc3        -DSL-TEDAIREAEDDWIDN-----------GGQNSSADELDMSY----------------ETLLAGVLIGF-FGGAIACYFLWERTMFS
DscC        PDTMPTGAELRELEDRWMDEGSSAMAAGVPGGGEGISFADDDGGF-----GA---GSRGAMDDMLWGAVMGFFWPVGCAMWLRREEGVWS
NCU11245    PDTMPSPDTLRSMEDAWIDNNHGGV------GGPGMVESDDGSGMVPEPIGAETDGFNGMVDTLVKGMFIGFVFPLGAIAWLLREEGMLS
             *:: :   :*. ** *:*:           ** .    : . .                 : :: * .:** :  .   :: .*  : *

Dsc3        LRMQLSILVGIICNFAYGLLHSYRW--
DscC        WRKGLAVFVGVVINVAFGAMRIMN---
NCU11245    KRWRVSIAFGVVFGFGIGLLRVFSSDQ
             *  ::: .*:: ... * :.
```

D

```
Dsc4        -----------------------------------------------------------MDTIVLDQRGE-------------VFSF
DscD        ----------------------------------MTTPDAFRDAEYIADVSGISYGNVANLTEQDANVVPRENERMRKLQLAAKVAFIDRL
NCU02676    MNDDSEPSFAARQQEQEQQQQQQQQQQQHIPPHNANEDGSRRNDNHERSTELN-GSAHDDNDDDDDQTSREEK--RKLGLKKKLQFMSHL
                                                                   *     .  :              :  :

Dsc4        FRSLDMLCYAIIAQQYFQDPTVLLLLLKVFVQLSYLTPK--PFSQLNALPLFYPLLLNFLISLMVRMFFNLPTAGESLDGYLYGGSIINF
DscD        LRDLDILIYCELSALYYMDCSVILFAIRAIVQLIFFTPKAPPFDPTRNQPFIGAIFVSNIFCMIFHNFFTHPEASEATRGYLHGGLLIDF
NCU02676    QRNLDMIYFAYLCTLYYMECSFLRLFVRIIPHFMFITPKEGPVLLPAERPHVFAILGPNLICILAHIISAPPVAGEATRGYLHGGVIVDF
             *.**:: :. :.  *: : :.: : :. : :: ::***  *.        * . .::   ::.:: . :   * *.*:  ***:** :::*

Dsc4        IGEKNESSRIDFITSDLVLFCLQIFMALILI----------------------ASNKRPTQASHIQASQSGLSNVDGDEEPSDLITEDSR
DscD        IGQKAPISLFRLFLLDFLVLILDLVMLGLIVERVK------------------TTGQTSTTSTEILR----VQDHDSEERGVHRTRPESR
NCU02676    VGQKPPTTRLGPLFFDAVILAIQCLMLAIHTEREKLRKVVNPSLQPLWAGPGQVGQQTGASGSDGVRQPETSQTLDAEERGVNREDEMAL
            :*:*   : :  :  * ::: :: .*  :                           .  :  : .:             .  *.:*        :

Dsc4        DTQQGQRQEDLQRQLENERSRLIARFSHSLY---SFQHGLSFGSPQNRNTPLQRQSADSYSTPVCIEVDSEDWKDLVWKSQYATENANTN
DscD        SSVVGAELDETDGHITRANAGVGEQAEHTQLLADPSEDGHTPGAKNSHPLDAFSSGEAVIMNLGLFDVIRDQWKYSTTAPPARTSSYIPS
NCU02676    GDGEGT---ETDRLVAESRGGQGQDDEQRL------GSNYSPASAGIDLFDVMRSGNASVGSFHPVHAIRTVGNDYQTAAAYTVQSIGFH
            .   *    : : :   ..        .:              . : .:          ..     .   . .      :     .   ...

Dsc4        SINNSPLSSNT---TGVPNSVLTNPI
DscD        DQTAAFLRARFGLQVGPDGRVQRIES
NCU02676    AAAVAGNRGVRGI-AGITNRAARRGI
               :   .    .*  . .
```

E

```
Sre1        ---MQSSIPSV------SVSVASP-AMETPTKASPDSKSPNSVGAIPSSSP------LASSTKASTSTPFVENCSNLLCDLASIVEDSPP
SrbA        -----MSTPGI--GG--DFQLFSP--------------------------------LESTRRISQGNSLS------------VDQSST
NCU04731    MADPDADSPGRFQGGLDSFDISTPLQLDEPTLARHGSLNANVNDAVTNSDPTSIPSSLADTSRASTGGGPTRQYPNHSPPIG--HHQQPN
                  . *.        ...: :*                                      * .: . * .             :..

Sre1        TLTNTSLSP-------HSFSLSD---MESSMSNWLNPFAFDNTMNSAPPLFTS-----TNMGSPNSLENSTNPLLSNCG-----SPNSFQ
SrbA        DVASQDWTQWMRWDDEQAFPETA---NASPSSPF--DLAFISPSASSGREASD------AMHKDFSPDISLDFKSPSLG-----------
NCU04731    AYHGNGISPQQQYQQQQQQQQAFDFNQDNISNW--DLR-DSPPFNSGPDFNNFGQVPHGLNIPQSWDLPVHFSINSQGLQQQGQPGQQQ
              . . :          :             .  * :   :   ..  .:    ..        :    * : .       . *

Sre1        NETF----------TGPSLNEFDADDKIQRQMKILHSVDTIDPSTVQNYPSADMSNPEVKLKTEEII--TPMDTTCKPEPSAKKIKLSPS
SrbA        ---FF---------PGGDLNTNVSPQPDHVGAGSLSVHSN-----------------------------SPLSSIG--------------
NCU04731    QGQFYPDTTGAGIDPGIYAAALSIPSPQQQSAEPISVRTNINPNIRHKLTPAQQERLKTIAMPPHLQYHSPKSAGSPDSNASMGHDKGSA
               *          .*         .  :     :    .                             :* .:

Sre1        SEDSCSIPETLPFSAPKSRGSLSPSETPDFVAGPGGKPKKTAHNMIEKRYRTNLNDRICELRDAVPSLRAAAALRCGN--SLDDEDLGGL
SrbA        -----------ASRKRKTGSDDDGSTMTSMFKAKQAPSKKRAHNVIEKRYRANLNEKIAELRDSVPSLRASYKQANGN--SGDDDD-DGV
NCU04731    SSPDGLEPSKPNSRKRKSSDEVDEDDDDDDLDGHQ-PVKKTAHNMIEKRYRTNLNDKIAALRDSVPALRIMSKSARGEDTTEDREELHGL
                        *: .. .  .    . . .     ** ***:******:***:.*. ***:**:**        *:  : * ::  *:

Sre1        TPARKLNKGTILAKATEYIRHLEAKNKELQKTNKQLSDRLAFYE--------------DP----------SMAPPSNDTRAVNSVNVVSS
SrbA        TSASKLNKASILSKATEYIRHLEIRNKRLEEENTALKIRLRQLDKAADQIVTSAASVSSPSDCTVSTESGASSSPSVFSHAEDVPS----
NCU04731    TPAHKLNKATVLSKATEYIRHLEKRNNRLIEENNSMHARIAAFEKLF--MAGAMTGMTNP----------MQAPPTPIQYPQDVAAFMST
            *.* ****.::*:********** .*: * : *. :   *:    :             .*             :.*:    . :

Sre1        SDYSVHQSSRP-------NLTQRAFTSPTLNT------------------------------------------MGRTALNGMVGLGL
SrbA        -DHSPTSSHPPEGLIKVPDAWKRMRAAGSNES--PY-------------SQSYIQYKKTDSHSSQSG-GGRMRSHLPNKYMLGALAGLMV
NCU04731    PMQTPRGPDPP-GLIPIPDDMKRILSAQQMNAGRPYPVGPPAIQPGFVANPGVIRRQQIQQQQQQHQMQGRWNPYL-GKMMVGSLAGLML
                :   .  *        :  :*  ::   ::                                          ..  :..:.** :

Sre1        FN-YFGNDSSQSVY---GLFALPP-------------------FLMSPFTGTVLFNMLKIGVVLLGLFYLLHDNSLFKGFKGEKKSKVST
SrbA        LE-GLGTEKKTEST-AKGLLAVPLNLLNRVQLPSEVYSSAAFQYFWSSWHARAISHFLMLAILVVGSAF-----IVFVYLFNSDPRRQYS
NCU04731    VEAAMEQEASNETTEGRGLYALPLQLIGSFIRSSHLSVGG---YYISAIDIIAKLKLLLLGALLVWTIF---PQSFFDAPKSEKPKGPTS
            .:  :  : . .      ** *:*                   :  *.    .  ::* :. :::   :       .*     ...    :

Sre1        RSSMSP----SSILFRK----TVFEKYCLLDHS--TSTISLFFGLLIFTLKSAYGYLTHRLSALYTSSENWVYSEQQLAEVRNMEKLLDA
SrbA        ASKVAPDVTLSSCNFRRQAWLTSIQRVGVPRHRFFHEWYVVTSRCFEYVLRCLLGWKLYSLVTGVT-------EEDEKGRVKTWDIAIDA
NCU04731    AVQAVPSLA-SPIHVRRQAWLTAIQTVWVPRHNFLLEATALLLKTLKFTLRNVFGVGTYLWMTGLS-------EEQEAARVKAWTIALDA
              .  *    *.  .*.    * ::   :  *     .   :    : :.*.    *    :    :   :        .*:: . *.     :**

Sre1        QLMGGDAKVDRLRLLMVFASSFSLPPSSHTCALQAMYCQLIFS--------NTSVPSAIVSKCVAFFWNAAKKQHS--KSSVHAELRELP
SrbA        QLAGGDAEISKSRLVLTIFAAGTLPRSPMRMMLKALHCRILMWRVGEPGSWTFNVSNDVARSLARYQWDLARKMNAALPKDHP---DSLP
NCU04731    QLAGGDVEINRSRLTLTLLASGTLPDTPHRLMLKALHTRVLLWQLSS----KFSLANLPAAKIARSRWNEARQLNMILQRTSTSPEDVLP
            ** ***.::.. ** :.: :: :** :.    *:*:: .:::           . .:.. . . .    *: *.: :              **

Sre1        ECTANLIENSHADDVFSPNMVERLWVLA---KCTRDSAQMSDSIISSLSDVLVLSPLEVLASWYAADLLDALLMESL-----------SR
SrbA        SHLATLLKID-CDDVMIDTIIQRAANLTWNRPTQEGTDDDEALLDVVEEDPAIQSSLDALAAWWSSHLLQGALLRYFEASSGGPDAKKSR
NCU04731    EHLATLLEQD-CDDVLNNSIVQRAHNLAFNRPTAFNLVDLTDGMNIVVEDPAVRSPMDAVAAWYSSSILQRVLLTSLARAQDG--ITDAT
            .  *.*:: . .***:  .:::*   *:        .  :     :     .*  : *.::.:*:*::: :*:   *:  :           :

Sre1        KVEISEIEEIISLCPKNSSIIRHALLAKLVLFPENTADSLNEVLAAYKNTL--DLCSQDKRKQSSVLKINLSKLFTL------HSCLSLA
SrbA        NVFKSKIKLALDVAPQPSAAHTRALVMMAVFFERDRVANIGSVLAA----LPKEKGKNKQNQASNFLDSSLPISVREEISTAVRCAMIAA
NCU04731    QEIIDEIALAAKIAPIGSNAYLRALVARAVLVSEKRGGSIAAALKASDPSLNPDKHPEYGRGVPPLIDSPMAQIVPD------HDALMAL
            :    .:*     .:.*   *    .**:    *:.  .   .:  .* *     *  :    :  .   . .:.  :.  .         . .:

Sre1        LQRLGYGDVSK-----------RMYQEIFVPDSD----ADITPLSFIISWTALNTF----------------------------------
SrbA        IFNARATGDTSLPATFTVEKAIHWFNRLPLDPVE------LTLLEFAAVYHLLHILASDIDYLASSDSSAPPSPMSKASDMLSSSSDDGE
NCU04731    QYAMRIAHLQKFPD--PPKQAIAFLNSILPGGTDQPKHEGMSLLAFTAAYCLMELL----------------------------------
                      .                : :      :       :: * *   :  :  :

Sre1        -----APICTSPKENDVVEKMAMYVRTAIGTLKIQDL-----KLSRKLINSCIDIGSRL-----------QEDLGYVSSA----------
SrbA        DGASQRNNNIIPHPMPNLGRVASELIYWARNAYNPAFYGFTSNLVKVIETECTSLCQTAGVHVADYSCVQEEKSKAKQAIDSKRRFAGGN
NCU04731    ----ERHTVCREQCSGALERLAGALRRWVGSPQGEQV-GLDGKMRQRMTDRCSSIIKSV--------IGMENDPGYGSMSDCEENEEGGC
                         :     : .:*  :       .       .      :: . :    * .: .                ::.     .

Sre1        -------------
SrbA        EEASDNLLLSDES
NCU04731    -------------
```

Figure S2. Dsc protein complex sequence alignments. The Dsc1-4p and Sre1p from *S. pombe*; and the DscA-D and SreA from *A. fumigatus* were aligned with their nearest homologs in *N. crassa* using the MUSCLE multiple sequence alignment tool. Conserved domains as identified by the BLASTp algorithm are as follows: **(A)** Alignment of Dsc1p and homologs: RING domain (zinc finger domain associated with mediating protein-protein interactions and ubiquitin ligase activity), red text; **(B)** Alignment of Dsc2p and homologs: UBA domain (ubiquitin associated domain, a novel sequence motif found in several proteins having connections to ubiquitin and the ubiquitination pathway), red text; DUF1751 domain (domain of unknown function found in eukaryotic integral membrane proteins), blue text. **(C)** Alignment of Dsc3p and homologs: DUF2407 domain (fungal domain of unknown function related to the ubiquitin domain), red text; DUF2407_C domain (fungal domain of unknown function), blue text. **(D)** Alignment of Dsc4p and homologs: DUF1746 domain (fungal domain of unknown function), red text. **(E)** Alignment of Sre1p and homologs: HLH domain (helix-loop-helix domain, found in specific DNA-binding proteins that act as transcription factors), red text; DUF2014 domain (domain of unknown function, found at the C-terminal of a family of ER membrane bound transcription factors called sterol regulatory element binding proteins (SREBP)), blue text.
