# Supplementary material for: Deletion of homologs of the SREPB pathway results in hyper-production of cellulases in Neurospora crassa and Trichoderma reesei
Source: Biotechnol Biofuels. 2015 Aug 19;8:121. doi: 10.1186/s13068-015-0297-9 (PMC4539670; doi:10.1186/s13068-015-0297-9)
Supplement: Supplementary file 4 — Additional file 4: Figure S3. Deletion of Dsc E3 ligase complex and SREBP1 component homologs showed a short aerial hyphae and resulted in higher cellulase production. [file 13068_2015_297_MOESM4_ESM.pdf]

**Figure S3**

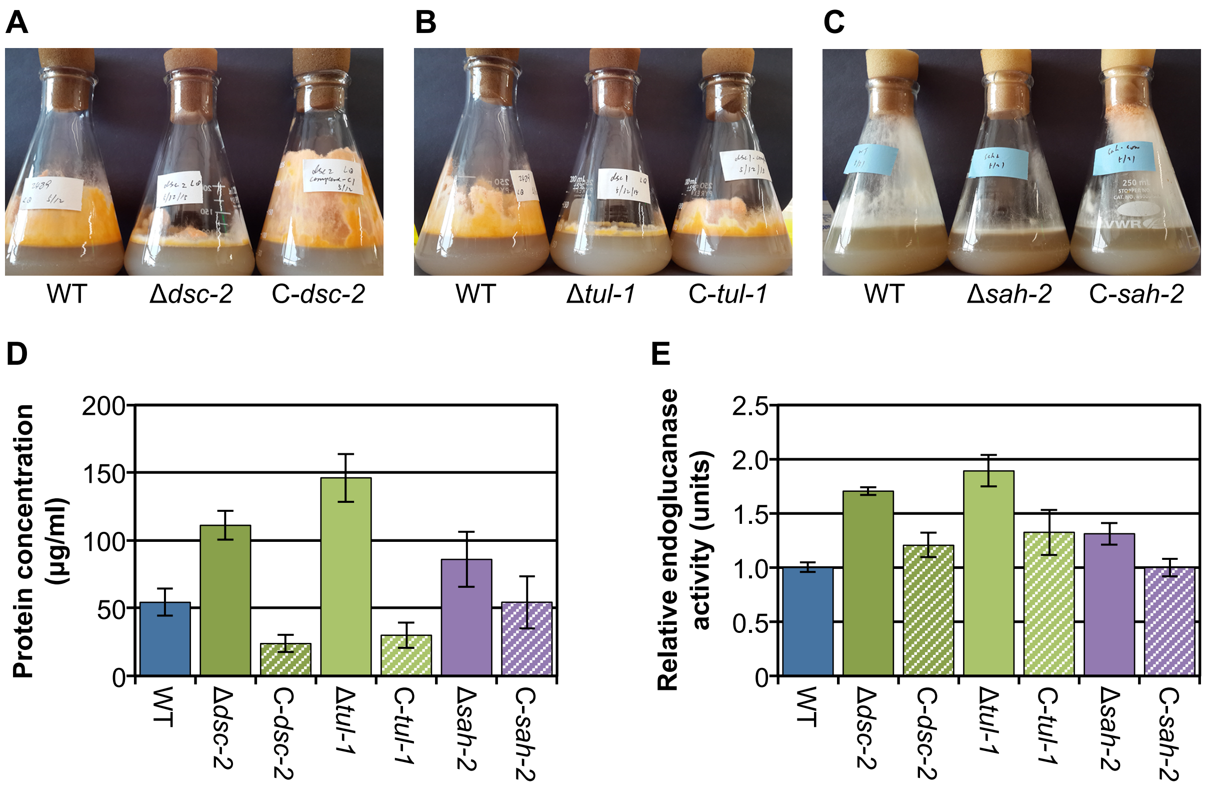


**Figure S3. Deletion of Dsc E3 ligase complex and SREBP1 component homologs showed short aerial hyphae and resulted in higher cellulase production. (A-C)**: Growth of WT, Δ*dsc-2* **(A)**, Δ*tul-1* **(B)** and Δ*sah-2* **(C)** and the complementation strains (C-*dsc-2*, C-*tul-1*, C-*sah-2*) on sucrose agar growth medium. Each deletion strain showed reduced aerial hyphae as compared to WT and complemented strains. **(D-E)**: Total secreted protein **(D)** and endoglucanase activity **(E)** levels in Δ*dsc-2*, Δ*tul-1* and Δ*sah-2* mutants compared to WT and complemented strains. Conidia from the indicated strains were inoculated directly into Avicel growth medium (120 hr). Statistical analysis for protein and enzyme activity levels relative to WT within each condition (n = 3) were performed as indicated in Figure 1. Error bars indicate standard deviation.
