# Supplementary material for: Deletion of homologs of the SREPB pathway results in hyper-production of cellulases in Neurospora crassa and Trichoderma reesei
Source: Biotechnol Biofuels. 2015 Aug 19;8:121. doi: 10.1186/s13068-015-0297-9 (PMC4539670; doi:10.1186/s13068-015-0297-9)
Supplement: Supplementary file 5 — Additional file 5: Table S2. Primers used in this study. [file 13068_2015_297_MOESM5_ESM.docx]

**Table S2. Primers used in this study.**

|  | **Primer**  **name** | **Primer sequence**  **(5' to 3')** |
| --- | --- | --- |
|  |  |  |
| **Primers used qRT-PCR study** | | |
|  | qRT-actin-F | TGATCTTACCGACTACCT |
|  | qRT-actin-R | CAGAGCTTCTCCTTGATG |
|  | qRT-cbh-1-F | ATCTGGGAAGCGAACAAAG |
|  | qRT-cbh-1-R | TAGCGGTCGTCGGAATAG |
|  | qRT-cbh-2-F | GAGGATCGCCAGAACTGC |
|  | qRT-cbh-2-R | GCACTGGGAGTAGTAAGAGTTG |
|  | qRT-gh5-1-F | TGAGTTCACATTCCCTGACAC |
|  | qRT-gh5-1-R | GGGTGAGGTTGGTGAGATAG |
|  | qRT-gh3-3-F | CGAGCCGATCCATCCTTCTCATCA |
|  | qRT-gh3-3-R | ATTGTTCAGCGCCCCAGCCG |
| **Primers used in the generation of *T. reesei* deletion strains** | | |
|  | pSC-pyr4A-F | GGGAGACCGGCAGCGGCCGCGTTGCATGATAATGGACTGGACC |
|  | pSC-pyr4A-R | GTAAGGTAGCTCTCGGATCCGGCTGATGAGGCTGAGAGAGGCTG |
|  | pSC-pyr4B-F | CAGCCTCTCTCAGCCTCATCAGCCGGATCCGAGAGCTACCTTAC |
|  | pSC-pyr4B-R | CTTCTTTCTTCCCTTCCCTCCTCGAGGGTACTATGGCTTAGATGG |
|  | pSC-pyr4C-F | CCATCTAAGCCATAGTACCCTCGAGGAGGGAAGGGAAGAAAGAAG |
|  | pSC-pyr4C-R | AATTGGAGCACTGCGAGGGGCCTGCCTTGGGCAGCACCAGCAGC |
|  | pSC-pyr4D-F | GCTGCTGGTGCTGCCCAAGGCAGGCCCCTCGCAGTGCTCCAATT |
|  | pSC-pyr4D-R | CCAGTCCATTATCATGCAACGCGGCCGCTGCCGGTCTCCCTATAG |
|  | pSC-SM-F | CAGCGAGAGCCTGACCTATTGCATC |
|  | pSC-SM-R | CAGGACATTGTTGGAGCCGAAATC |
|  | pSC-tul1A-F | TATAGGGAGACCGGCAGCGGCCGCAAGGACAAGGTCCAGATTGCAAAG |
|  | pSC-tul1A-R | GATATTGCGACTTTGGGGGTGAGGCTGGAGCCGCGGAATCGGGCTATG |
|  | pSC-tul1B-F | CATAGCCCGATTCCGCGGCTCCAGCCTCACCCCCAAAGTCGCAATATC |
|  | pSC-tul1B-R | ATACCGAATCTCTTCGACAGCCCACAACTGCATCCAAACCATCCTAC |
|  | pSC-tul1C-F | CATAGCCCGATTCCGCGGCTCCAGCGATTCGGCTAGTATTCACACACCAC |
|  | pSC-tul1C-R | GAAAATTGGAGCACTGCGAGGGGCCTTTCCGCTTCCCCATGACCACTCAC |
|  | pSC-tul1D-F | GTGAGTGGTCATGGGGAAGCGGAAAGGCCCCTCGCAGTGCTCCAATTTTC |
|  | pSC-tul1D-R | CTTTGCAATCTGGACCTTGTCCTTGCGGCCGCTGCCGGTCTCCCTATA |
|  | Tul1-P1-F | TAACCGCAATACCACGACAGTATC |
|  | Tul1-P1-R | CTTTGTTAACCGGGGCGAGGAGAC |
|  | Tul1-P2-F | GGAGCTCCCTGCCTACAAGGTCCTCTT |
|  | Tul1-P2-R | ACGAAAGCGCAGATGTTGAGGAAC |
|  | pSC-sah2A-F | CTATAGGGAGACCGGCAGCGGCCGCCAGCATTGAGAAAGTCGACAAAG |
|  | pSC-sah2A-R | CGATATTGCGACTTTGGGGGTGAGGGCAATGTTTCTCAGTTGTTCC |
|  | pSC-sah2B-F | GGAACAACTGAGAAACATTGCCCTCACCCCCAAAGTCGCAATATCG |
|  | pSC-sah2B-R | GTTGGTACCTTGGGGGAGCTTCGTGCAACTGCATCCAAACCATCCTACC |
|  | pSC-sah2C-F | TGCAGGAACAACTGAGAAACATTGCCGGCTGGCGAGCTCTAGAGAAGG |
|  | pSC-sah2C-R | GAAAATTGGAGCACTGCGAGGGGCCTAGACGGGCAAGATTCCCGCAGTGG |
|  | pSC-sah2D-F | CCACTGCGGGAATCTTGCCCGTCTAGGCCCCTCGCAGTGCTCCAATTTTC |
|  | Sah2-P1-F | GGACACACAGGATTCGAGCATGTCG |
|  | Sah2-P1-R | GCACATTGTCAACGTCAACATGGTGCCC |
|  | Sah2-P2-F | ATGCCCCCTCATCTTCAGTACGGC |
|  | Sah2-P2-R | CTTCGTCTCCGTATAGCGTCGTCG |
