# Supplementary material for: Deletion of homologs of the SREPB pathway results in hyper-production of cellulases in Neurospora crassa and Trichoderma reesei
Source: Biotechnol Biofuels. 2015 Aug 19;8:121. doi: 10.1186/s13068-015-0297-9 (PMC4539670; doi:10.1186/s13068-015-0297-9)
Supplement: Supplementary file 7 — Additional file 7: Figure S5. Generation of tul1 and sah2 deletion mutants in T. reesei and their corresponding hyper-production phenotypes. [file 13068_2015_297_MOESM7_ESM.pdf]

Figure S5

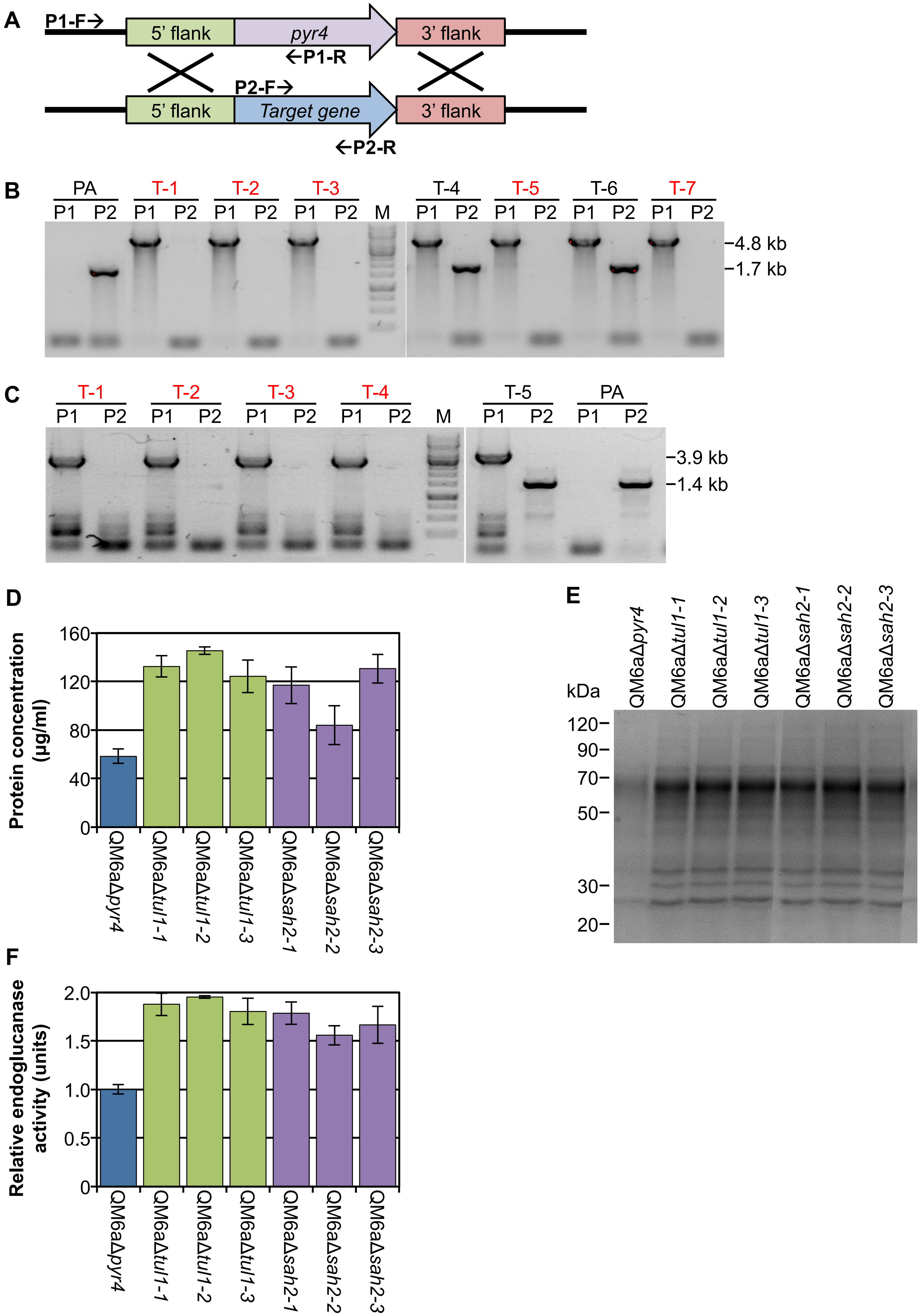
A
P1-F
5' flank
pyr4
3' flank
P1-R
P2-F
5' flank
Target gene
3' flank
P2-R
B
PA
T-1
T-2
T-3
M
T-4
T-5
T-6
T-7
P1 P2
4.8 kb
1.7 kb
C
T-1
T-2
T-3
T-4
M
T-5
PA
P1 P2
3.9 kb
1.4 kb
D
Protein concentration (μg/ml)
160
120
80
40
0
QM6aΔpyr4
QM6aΔtul1-1
QM6aΔtul1-2
QM6aΔtul1-3
QM6aΔsah2-1
QM6aΔsah2-2
QM6aΔsah2-3
E
kDa
120
90
70
50
30
20
QM6aΔpyr4
QM6aΔtul1-1
QM6aΔtul1-2
QM6aΔtul1-3
QM6aΔsah2-1
QM6aΔsah2-2
QM6aΔsah2-3
F
Relative endoglucanase activity (units)
2.0
1.5
1.0
0.5
0
QM6aΔpyr4
QM6aΔtul1-1
QM6aΔtul1-2
QM6aΔtul1-3
QM6aΔsah2-1
QM6aΔsah2-2
QM6aΔsah2-3

Figure S5. Generation of *tul1* and *sah2* deletion mutants in *T. reesei* and their corresponding hyper-production phenotypes. (A-C) Genotyping of *T. reesei* transformants. **(A)** Schematic drawing of the primer positions for analytic PCR of the deletion loci. **(B-C)** PCR analysis of Δ*tul1* transformants **(B)** and Δ*sah2* transformants **(C)**. M indicates DNA ladder marker, PA indicates parental QM6aΔ*pyr4* strain, T indicates transformant strains. Transformants highlighted in red indicate positive transformants. **(D)** Total secreted protein. **(E)** Endoglucanase activity. Conidia from the indicated strains were inoculated directly into growth media containing Avicel and cultured for 7 days. Statistical analysis for protein and enzyme activity levels relative to the parental QM6aΔ*pyr4* (n = 3) were performed as indicated in Figure 1. **(F)** SDS-PAGE analysis of total secreted protein 6 days after inoculation, with equal volumes loaded.
